# Gut microbiota from persons with attention-deficit/hyperactivity disorder affects the brain in mice

**DOI:** 10.1186/s40168-020-00816-x

**Published:** 2020-04-01

**Authors:** Anouk C. Tengeler, Sarita A. Dam, Maximilian Wiesmann, Jilly Naaijen, Miranda van Bodegom, Clara Belzer, Pieter J. Dederen, Vivienne Verweij, Barbara Franke, Tamas Kozicz, Alejandro Arias Vasquez, Amanda J. Kiliaan

**Affiliations:** 1 Department of Anatomy, Donders Institute for Brain, Cognition & Behaviour, Preclinical Imaging Centre PRIME, Radboud University Medical Center, Geert Grooteplein noord 21, 6525 EZ Nijmegen, The Netherlands; 2 Department of Cognitive Neuroscience, Donders Institute for Brain, Cognition and Behaviour, Radboud University Medical Center, 6525 EN Nijmegen, The Netherlands; 3 Donders Institute for Brain, Cognition and Behaviour, Radboud University, 6525 EN Nijmegen, The Netherlands; 4 Dept. Agrotechnology and Food Sciences, Wageningen UR (University & Research), 6708WE Wageningen, The Netherlands; 5 Department of Psychiatry, Donders Institute for Brain, Cognition and Behaviour, Radboud University Medical Center, 6525 GC Nijmegen, The Netherlands; 6 Department of Human Genetics, Donders Institute for Brain, Cognition and Behaviour, Radboud University Medical Center, 6525 GA Nijmegen, The Netherlands; 7 Department of Clinical Genomics, Mayo Clinic, Rochester, MN 55902 USA

**Keywords:** ADHD, Microbiota, Behavior, Gray and white matter integrity, Functional connectivity, DTI, rs-fMRI

## Abstract

**Background:**

The impact of the gut microbiota on host physiology and behavior has been relatively well established. Whether changes in microbial composition affect brain structure and function is largely elusive, however. This is important as altered brain structure and function have been implicated in various neurodevelopmental disorders, like attention-deficit/hyperactivity disorder (ADHD). We hypothesized that gut microbiota of persons with and without ADHD, when transplanted into mice, would differentially modify brain function and/or structure. We investigated this by colonizing young, male, germ-free C57BL/6JOlaHsd mice with microbiota from individuals with and without ADHD. We generated and analyzed microbiome data, assessed brain structure and function by *magnetic resonance imaging* (*MRI*), and studied mouse behavior in a behavioral test battery.

**Results:**

Principal coordinate analysis showed a clear separation of fecal microbiota of mice colonized with ADHD and control microbiota. With diffusion tensor imaging, we observed a decreased structural integrity of both white and gray matter regions (i.e., internal capsule, hippocampus) in mice that were colonized with ADHD microbiota. We also found significant correlations between white matter integrity and the differentially expressed microbiota. Mice colonized with ADHD microbiota additionally showed decreased resting-state functional MRI-based connectivity between right motor and right visual cortices. These regions, as well as the hippocampus and internal capsule, have previously been reported to be altered in several neurodevelopmental disorders. Furthermore, we also show that mice colonized with ADHD microbiota were more anxious in the open-field test.

**Conclusions:**

Taken together, we demonstrate that altered microbial composition could be a driver of altered brain structure and function and concomitant changes in the animals’ behavior. These findings may help to understand the mechanisms through which the gut microbiota contributes to the pathobiology of neurodevelopmental disorders.

Video abstract.

## Background

The gut-brain axis (GBA) is a well-recognized bidirectional communication route between gut and brain [1]. One of the key modifiers of the GBA is the intestinal commensal bacteria in the gut, also known as the microbiota. The intestinal microbiota is a complex ecosystem that comprises more than 1000 different species [2], which can be influenced by numerous factors, including diet, antibiotic usage, lifestyle, and host genetics [3, 4].

The role of gut microbiota in host physiology and health has been well established [5]. An increasing body of literature recognizes the influence of the microbiota on neurodevelopment and brain function as well [6, 7]. Studies in animal models have reported an essential role for the microbiota in fundamental neural processes such as neurogenesis, myelination, and microglia activation [8]. In germ-free (GF) animals, affected hippocampal volume and neurogenesis are observed, in addition to hypermyelination of axons in the prefrontal cortex [9–17]. Moreover, modulation of the gut microbiota via diet induces diet-dependent global changes in white matter structural integrity in rat models [18]. Perturbations in microbial composition have also been associated with differences in behavior and cognition [19]. Particularly, early life disturbances of the microbiota can influence neurodevelopment, possibly resulting in psychiatric disorders later in life [20].

An increasing number of studies report possible roles for the microbiota in anxiety and social behavior [11, 12, 21–23], which have been confirmed by a limited number of human studies [24–27]. These observations suggest that the microbiota may play a key role in the development or manifestation of many psychiatric disorders [28]. Several psychiatric disorders, including autism spectrum disorder (ASD), major depressive disorder (MDD), and attention-deficit/hyperactivity disorder (ADHD), are associated with differences in the gut microbiota [29–31]. Previous research has demonstrated changes in the gut microbiota of children with ASD compared to healthy controls, including a higher *Firmicutes*/*Bacteroidetes* ratio and a higher relative abundance of *Clostridia* species [30, 32], whereas MDD has been associated with an increased microbiota alpha diversity [33]. For ADHD, several studies, by us and others, have identified differences in microbiota composition between individuals with ADHD and healthy controls [34, 35]. An increase in the genus *Bifidobacterium* was observed in individuals with ADHD, associated with a significantly enhanced predicted biosynthesis potential of the dopamine precursor phenylalanine [34]. Additionally, dietary interventions in ADHD can be beneficial in subgroups of children with ADHD [36–39]. Similarly, for ASD, an improvement in symptoms has been observed after intervention. Here, antibiotic treatment alleviated anxiety in children with ASD [40], and treatment with vancomycin, an antibiotic used to treat infections with *Clostridia*, resulted in significant short-term improvement in neurobehavioral symptoms in children with autism [41]. Following the discontinuation of vancomycin, the behavioral improvements largely waned.

In the current study, we aimed to investigate the impact of human microbiota from individuals with ADHD on brain function and/or structure and behavior in mice. We hypothesized that the gut microbiota of individuals with ADHD, when transplanted to mice, would modify brain structure and/or function as well as influence behavior, biological domains that are also impaired to various degrees in neurodevelopmental disorders in humans. To test this hypothesis, we colonized young, germ-free mice with microbiota collected from male individuals with ADHD (from now on called “mice^ADHD^”) or microbiota from age-matched healthy controls (“mice^control^”). We subsequently performed structural and resting-state functional magnetic resonance imaging (fMRI) and analyzed different aspects of behavior in the microbiota-colonized mice. Extending insights on the impact of ADHD microbiota on brain phenotypes and behavior might increase our understanding of disorder etiology and ultimately open a window of opportunity for the development of novel treatment approaches for ADHD by targeting the microbiota.

## Results

A summary of significant results is given in Table 1.

**Table 1:** Overview of significant results

Parameter			Increase or decrease in mice^ADHD^
Microbiota		Beta diversity	↑
	Genus level	g_Porphyromonadaceae_uncultured; g_Clostridiales_unknown; Anaerostipes; Coprococcus_2; Epulopiscium; Fusicatenibacter; Lachnospiraceae_ND3007_group; Roseburia; Eubacterium_fissicatena_group; Eubacterium_xylanophilum_group Ruminococcus_gauvreauii_group; Ruminococcus_gnavus_group; Ruminococcaceae_UCG-004; g_Ruminococcaceae_uncultured	↑
		g_Bacteriodales_unknown; Coprobacter; Parabacteroides; g_Gastranaerophilales_unknown; Catabacter; Eubacterium; Eisenbergiella; Lachnoclostridium; Eubacterium_rectale_group; Anaerotruncus; Ruminococcaceae_UCG-014; Ruminococcus_1; Eubacterium_coprostanoligenes_group; Dielma; Holdemania; g_Enterobacteriaceae_unknown; Escherichia-Shigella	↓
	Family level	Clostridiales unknown	↑
		Porphyromonadaceae; Bacteriodales; Gastranaerophilales_unknown; Christensenellaceae; Eubacteriaceae; Ruminococcaceae; Enterobacteriaceae	↓
	Order level	Gastranaerophilales; Enterobacteriales	↓
	Class level	Melainabacteria; Gammaproteobacteria	↓
	Phylum level	Proteobacteria; Cyanobacteria	↓
Open field test		Center duration	↓
		Corner duration	↑
Diffusion Tensor Imaging	Fractional anisotropy	Right hippocampus	↓
		Left hippocampus	↓
		Right internal capsule	↓
		Right optic tract	↓
	Mean diffusivity	Right hippocampus	↑
		Fornix	↓
	Axial diffusivity	Right auditory cortex	↑
	Radial diffusivity	Right hippocampus	↑
		Left hippocampus	↑
		Right internal capsule	↑
		Corpus callosum	↑
Resting-state fMRI		Between motor cortex (M1) and visual cortex (V1)	↓

↑, significant increase; ↓, significant decrease

### Differences in microbiota composition between mice^ADHD^ and mice^control^

**Fig. 1:**
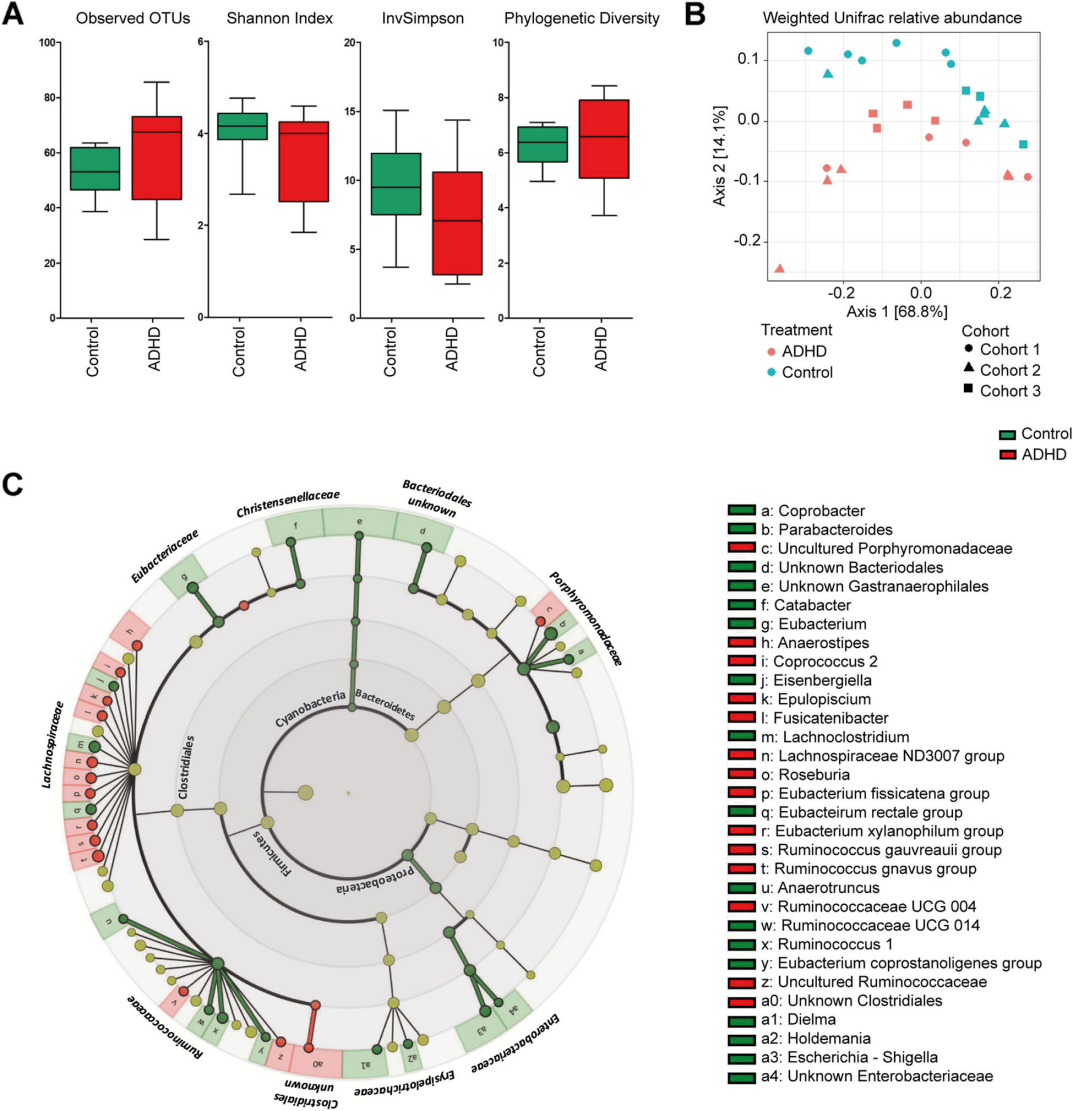
Microbial analyses. **a** Comparison of diversity between microbiota samples from mice^ADHD^ and mice^control^ using alpha diversity measures Observed OTUs (richness), Shannon Index, InvSimpson Index, and Phylogenetic Diversity. **b** Principal coordinate analysis (PCoA) plot of weighted UniFrac distances showing a clear separation in microbial composition between mice^ADHD^ and mice^control^. **c** Circular representation of the different bacterial genera between mice^ADHD^ and mice^control^ using LEfSe analysis (LDA score > 2.0, *p* < .05 unadjusted). Labels in inner circle represent phyla, and on the outer circle are the labels of the families

First, we assessed the microbiota composition of feces from mice^ADHD^ and mice^control^. The within-sample diversity (α-diversity) showed no significant differences between the mice^ADHD^ and mice^control^ (Observed: *t*_17.2_ = 1.181, *p* = .254; Shannon: *t*_19.4_ = − 1.731, *p* = .099; InvSimpson: *t*_25_ = − 1.781, *p* = .087; PD: *t*_15.9_ = 0.421, *p* = .680; Fig. 1a). Comparison of groups in terms of β-diversity showed a significant difference between mice^ADHD^ and mice^control^ (PERMANOVA *p* = .01). Mice^ADHD^ clustered separately from mice^control^ in the PCoA plot, as illustrated in Fig. 1b.

Linear discriminant analysis effect size (LEfSe) analysis showed that 31 genera differed in relative abundance between the two experimental groups (mice^ADHD^ vs. mice^control^ ; LDA score > 2.0, *p*_unadjusted_ < .05; Table 1 and Fig. 1c legend). These genera were part of the phyla Firmicutes (24/31), Bacteroidetes (4/31), Proteobacteria (2/31), and Cyanobacteria (1/31) (Fig. 1c, inner circle labels). In total, 14 genera were enriched in mice^ADHD^, while 17 other genera were more abundant in mice^control^. From the genera enriched in mice^ADHD^, 10 belonged to the family of *Lachnospiraceae* within the phylum Firmicutes. The overall phyla of Proteobacteria and Cyanobacteria were decreased in relative abundance in mice^ADHD^, and also the family of *Porphyromonadaceae* was present in lower relative abundance. Additionally, from the phylum of Firmicutes, the *Eubacteriaceae*, *Christensenellaceae*, and *Ruminococcaceae* families were less abundant in mice^ADHD^ (Fig. 1c, outer circle labels). The only family that was present in higher relative abundance in the mice^ADHD^, compared to the mice^control^_,_ was an unknown *Clostridiales*.

### Decreased structural integrity of both white and gray matter and reduced functional connectivity in mice^ADHD^

Diffusion tensor imaging (DTI) was used to assess gray and white matter integrity in several brain regions. Fractional anisotropy (FA) is a summary measure of microstructural integrity, and a higher FA might reflect increased white matter integrity. Mean diffusivity (MD) is an inverse measure of membrane density, and a higher MD might indicate decreased gray matter integrit y[42].

**Fig. 2:**
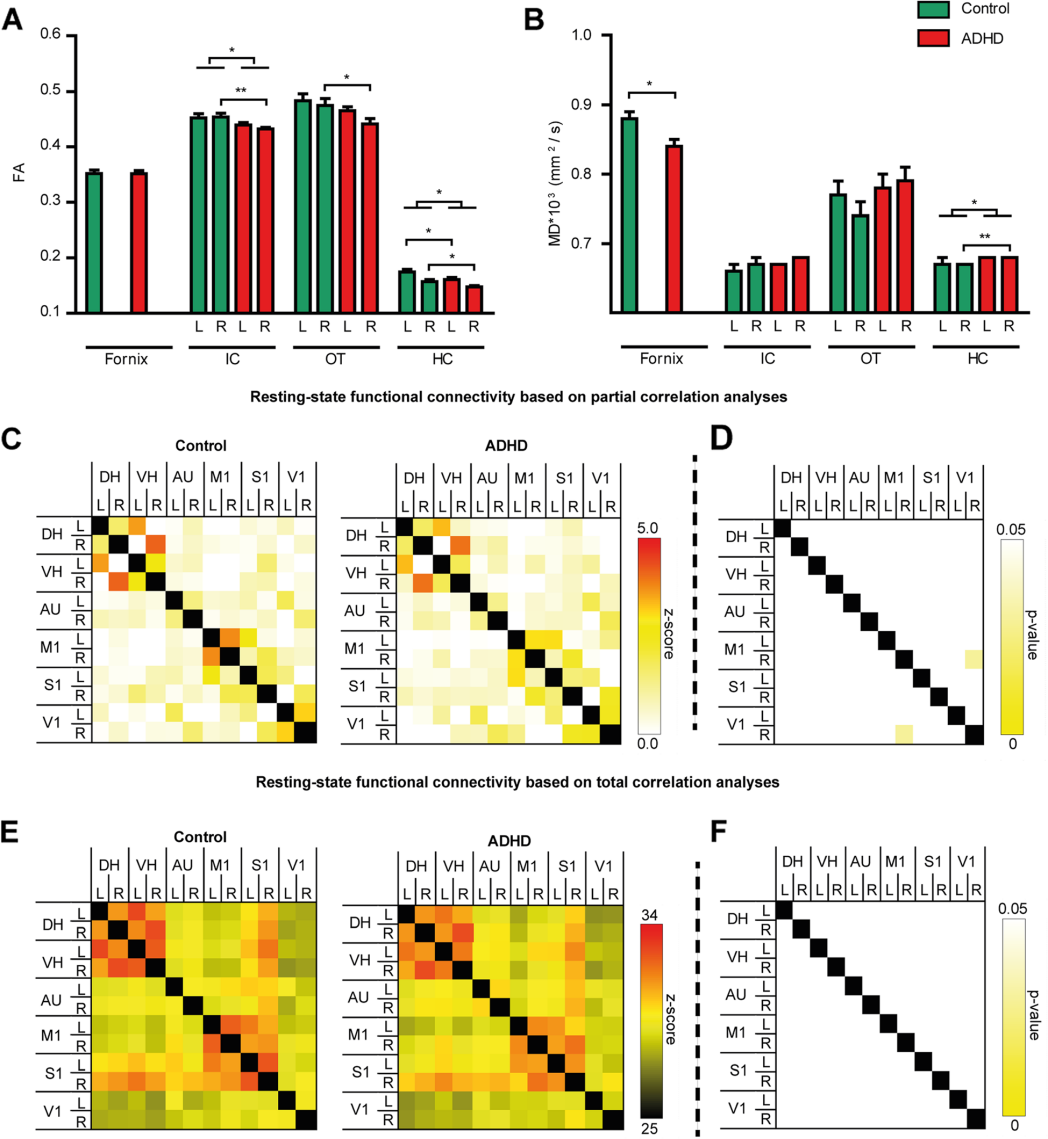
Effects of ADHD microbiota on brain structure and function. **a** Differences in fractional anisotropy between mice^ADHD^ and mice^control^ in the right internal capsule (*p* = 0.0007), right optic tract (*p* = 0.047), left hippocampus (*p* = 0.02), and right hippocampus (*p* = 0.047) were found. **b** Differences in mean diffusivity in the fornix (*p* = 0.044) and right hippocampus (*p* = 0.002) were found. Resting-state functional connectivity (FC) based on total (**c**, **d**) and partial (**e**, **f**) correlation analyses of 12 regions of interest (ROIs) in the mouse brain. Total (**c**) and partial (**e**) correlation matrices of control (left) and ADHD (right) mice. **f** A decreased al correlation analysis between the right motor cortex and right visual cortex (*p* = 0.03) was found in mice^ADHD^

The MRI data showed that mice^ADHD^ had a decreased FA in the left and right hippocampus (left: *F*(1,20) = 6.4, *p* = .020; right: *F*(1,20) = 4.5, *p* = .047). Lowered FA in mice^ADHD^ was also observed in the right internal capsule (*F*(1,20) = 9.2, *p* = .007) and right optic tract (*F*(1,20) = 4.5, p = .047) (Fig. 2a). Increased MD was detected in the right hippocampus (*F*(1,20) = 13.1, *p* = .002) of mice^ADHD^. An increased MD was also observed in the fornix of mice^ADHD^ (*F*(1,20) = 4.6, *p* = .044) (Fig. 2b). Mice^ADHD^ showed heightened radial diffusivity (RD) in the corpus callosum (*F*(1,20) = 4.8, *p* = .041), left and right hippocampus (left: *F*(1,20) = 5.3, *p* = .032; right: *F*(1,20) = 17.0, *p* = .001), and right internal capsule (*F*(1,20) = 7.3, *p* = .014). Finally, we observed an increased axial diffusivity (AD) in the right auditory cortex (*F*(1,20) = 4.6, *p* = .044) in mice^ADHD^ compared to mice^control^.

Because of the observed differences in FA and MD in the hippocampus, we also assessed hippocampal volumes. However, we did not find differences in hippocampal volumes between mice^ADHD^ and mice^control^ (*F*(1,23) = 0.07; *p* = .798).

Functional connectivity (FC) patterns, derived from resting-state fMRI (rs-fMRI) data, showed mice^ADHD^ to have a decreased FC in the partial correlation analysis between the right motor cortex and right visual cortex (*F*(1,14) = 5.9, *p* = .030; Fig. 2c, d). We found no effects in the total correlations (Fig. 2e, f).

### Higher anxiety in mice colonized with ADHD microbiota, but no memory deficits or impulsive behavior in mice

**Fig. 3:**
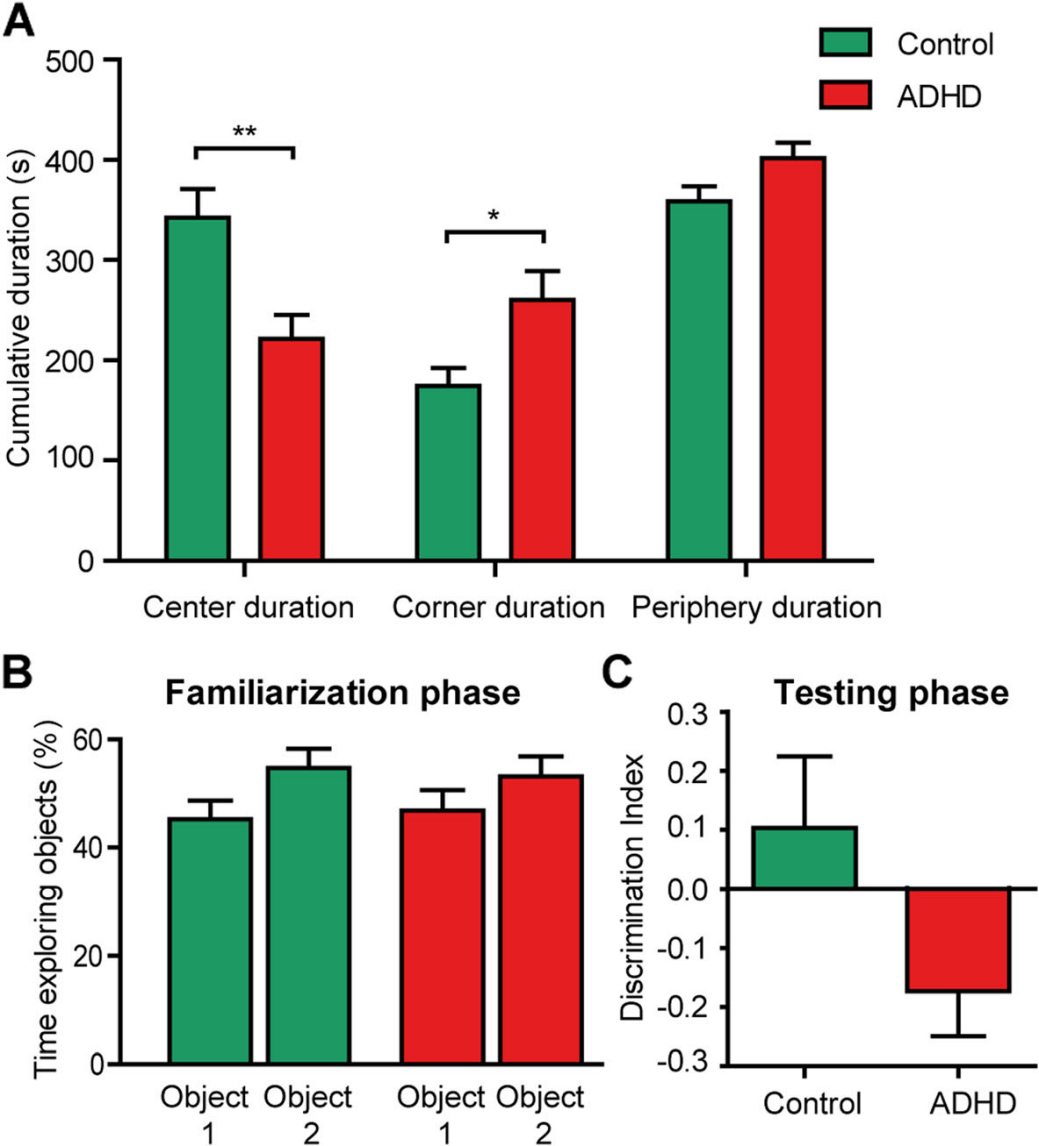
Effect of ADHD microbiota on behavior. **a** Time spent in the center (left), corners (middle), and periphery (right) of the open field. Mice^ADHD^ spent less time in the center (*p* < 0.004) and more time in the corners (*p* < .017) of the open field. **b** During the familiarization phase of the NOR test mice^control^ (green bars) and mice^ADHD^ (red bars) explored both identical objects equally. **c** Mice^ADHD^ showed a trend toward a lower novel object discrimination index (*p* = 0.063) during the test phase of the NOR

To explore behavioral effects of the human ADHD microbiota in mice, several behavioral tests were performed. We assessed general locomotor activity and novel environment exploration with the open field test (OFT). Mice^ADHD^ spent more time in the corners (*F*(1,24) = 6.6, *p* = .017) and less time in the center (*F*(1,24) = 10.4, p = .004) of the open field arena compared to mice^control^ (Fig. 3a). Groups did not differ in the frequency of entering the corners (*F*(1,24) = 1.8, *p* = .190) or center (*F*(1,24) = 1.7, *p* = .211). This suggests that the altered exploratory behavior in mice^ADHD^ reflects increased anxiety. Analysis of locomotion activity did not reveal any differences in velocity (*F*(1,24) = 0.4, *p* = .527), total distance traveled (*F*(1,24) = 0.2, *p* = .687), or any of the manually scored behaviors, like jumping, wall leaning, rearing, and grooming. Additionally, no differences in home cage activity during day or night were observed between the two experimental groups (Additional file 1: Figure S1).

During the familiarization phase of the novel object recognition (NOR) test, used to assess memory, all mice explored both identical objects equally (*F*(1,25) = .956, *p* = .338; Fig. 3b). Mice^ADHD^ demonstrated a trend toward a lower discrimination index compared to mice^control^ during the test phase of the NOR with a half hour interval between familiarization and test phase (F(1,25) = 3.8, *p* = .063; Fig. 3c), but not when an hour interval between the familiarization and test phase was used (*F*(1,25) = 1.98, *p* = .171; Additional file 1: Figure S2).

It has been proposed that burying of harmless objects, like glass marbles, reflects a form of compulsive or impulsive behavior [43]. Performing the marble burying test (MBT), we found no differences between the number of marbles buried by mice^ADHD^ (mean = 1.89; SEM = .91) and by mice^control^ (mean = 1.75; SEM = .61, *H*(1) = 0.56, *p* = .453; Additional file 1: Figure S3).

### Changes in microbiota are associated with anxiety and DTI measures in the hippocampus

**Fig. 4:**
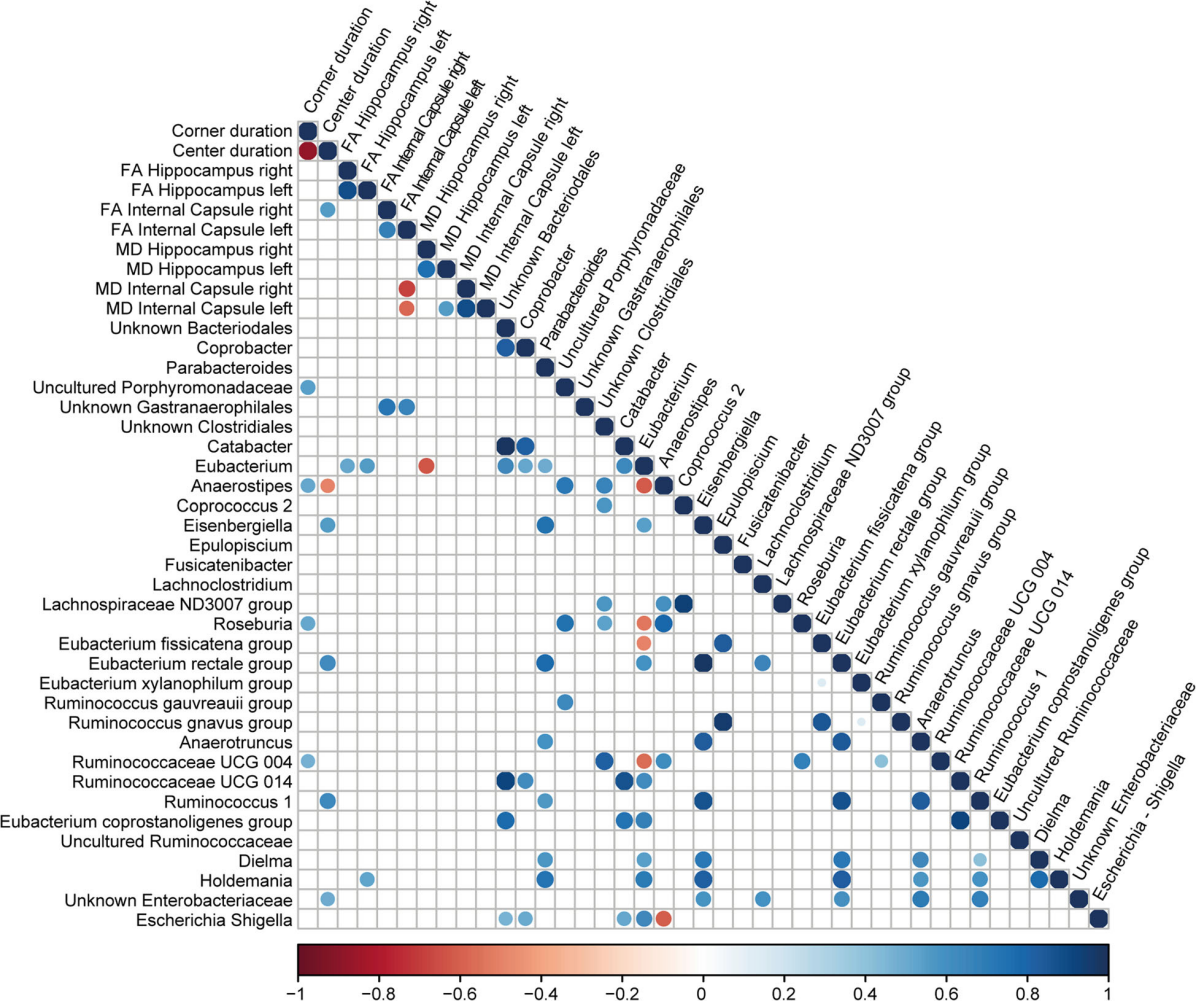
Correlations between bacterial genera and key neurobiological findings. Pearson correlations between relative taxa abundance and anxiety (center duration and corner duration in the open field) or DTI measures FA and MD in the hippocampus and internal capsule. All correlations presented were statistically significant (*p* < 0.01) with strong correlations indicated by large circles and weaker correlations by small circles. The colors denote whether the correlation is negative (red) or positive (blue)

We investigated, whether the genera that differed in relative abundance, described above and depicted in Fig. 1c, could be correlated to key neurobiological features (i.e., anxiety and DTI measures in the hippocampus and internal capsule). Figure 4 provides an overview of all observed correlations (*p* < .01).

The relative abundance of *Eubacterium* showed a positive correlation with the FA of the right and left hippocampus (FA; right: *R* = .564, *p* = .006; left: *R* = .586, *p* = .004) and a negative correlation with the MD in the right hippocampus (MD; *R* = − .559; *p* = .007). *Gastranaerophilales* unknown showed a positive correlation with the FA in the right and left internal capsule (right: *R* = .721, *p* < .001; left: *R* = .666, *p* = .001). Finally, we observed a positive correlation between *Holdemania* and the FA in the left hippocampus (*R* = .563; *p* = .006).

The relative abundance of *Anaerostipes* showed a positive correlation with anxiety (time spent in corners) in the OFT (*R* = .546; *p* = .005) and a negative correlation with the time spent in the center of the open field (*R* = − .512; *p* = .009). Additionally, the relative abundance of the *uncultured Porphyromonadaceae* (*R* = .555; *p* = .004), *Roseburia* (*R* = .541; *p* = .005), and *Ruminococcaceae UCG 004* (*R* = .515; *p* = .008) showed a positive correlation with anxiety (time spent in corners) in the OFT. The relative abundance of *Eisenbergiella* (*R* = .543; *p* = .005), *Eubacterium rectale* group (*R* = .626; *p* = .001), and *Ruminococcus 1* (*R* = .562; *p* = .003) showed a positive correlation with time spent in the center in the OFT.

## Discussion

Given the current knowledge on the association between the gut microbiota and neurodevelopmental disorders via the gut-brain axis [29–31], we hypothesized that the microbiota of individuals with ADHD, when transplanted into young GF mice, would affect brain structure and/or function as well as behavior. We show that mice^ADHD^ had an impaired structural integrity of both white and gray matter, and showed decreased functional connectivity between the right visual cortex and right motor cortex.

In the current study, we investigated if there were any structural brain differences in mice^ADHD^ compared to mice^control^ using diffusion tensor imaging (DTI). One of the brain regions selected was the hippocampus. The hippocampus is highly sensitive to aging, life experiences such as stress, and environmental factors like malnutrition and altered microbial composition [44–46]. The hippocampus is important for learning and memory, mood regulation, and neural plasticity [44]. It is therefore not surprising that impaired hippocampal function has been implicated in many neurodevelopmental and psychiatric disorders, including ASD, ADHD, and MDD [16, 47–50]. The DTI data revealed a decreased FA and increased MD in the right hippocampus, accompanied by increased RD and unchanged AD in mice^ADHD^. This is indicative of decreased myelination rather than axonal damage or degeneration [42]. A decreased FA and increased MD have been reported in children and adults with ADHD in several brain regions, including the internal capsule, but not in the hippocampus [51, 52]. However, a smaller hippocampal volume has been reported in ADHD [15, 16].

We also observed white matter alterations in the right internal capsule. The internal capsule is composed of afferent and efferent myelinated fibers of the cerebral cortex [53]. Abnormalities in the structure of the internal capsule have been implicated in neurodevelopmental and psychiatric disorders like schizophrenia, ADHD, and bipolar disorder [52, 54, 55]. We report decreased FA and increased RD in mice^ADHD^ in the internal capsule, indicative of decreased myelination in this region. This finding may be related to the observed decreased functional connectivity between the right primary motor cortex (M1) and the right primary visual cortex (V1) of mice^ADHD^ because the fibers of these regions course through the internal capsule [56]. Both the primary motor cortex and visual cortex are part of the visuomotor network [57]. Reduced visuomotor adaptation has also been reported in schizophrenia and in individuals with ADHD [58, 59].

We used various behavioral tests to investigate diverse aspects of mouse behavior that might be influenced by microbiota from individuals with ADHD. One of the tests that the mice performed was the OFT. In this test, we observed that mice^ADHD^ showed more anxiety than mice^control^, which was measured as time spent in the corners of the open field. Although this behavior is not specific to ADHD, many psychiatric disorders, including ADHD, MDD, and ASD, are highly comorbid with anxiety [60–62]. Altered anxiety-related behavior is often found after microbiota manipulations, and this behavior can also be caused by fecal transplantation [19]. This suggests that components of the gut microbiota are able to regulate anxiety. We found no differences in motor activity between groups in either the OFT or in home cage activity during active and inactive phase of the mice. Additionally, no apparent effects were seen in the MBT assessing compulsivity/impulsivity nor in the memory assessing NOR task.

Previous studies have examined and discovered some differences in the microbiota composition between individuals with ADHD and controls [34, 35], although transplantation of the microbiota of individuals with ADHD into mice is novel. Therefore we investigated the microbial composition within and between samples and on different levels in the current study. The overall microbial composition, after transplantation, revealed a clear separation (beta-diversity) between microbiota from mice^control^ and mice^ADHD^ (Fig. 1b). We did not observe, however, any significant differences in the alpha-diversity indices. Furthermore, in the global microbial composition, we found a difference in microbial community composition, with 31 genera showing different relative abundance between the animal groups. Most of these genera (23 of the 31) are also present in our human donors (ADHD and/or control), though we cannot be sure about the uncultured or unknown genera (6 out of 31). This means that our study has a translational value, especially as the microbial composition of both the original human samples and the mouse samples cluster per experimental group (ADHD and control).

One of the genera we found to differ between our groups was *Lachnoclostridium*. It appeared to be decreased in relative abundance in mice^ADHD^. The relative abundance of *Lachnoclostridium* was also found decreased in children with ASD [63] and in ADHD patients compared to controls [34, 35]. Going up to the phylum level, the differentially abundant genera in our study belonged predominantly (24 out of 31) to the phylum Firmicutes, and families *Lachnospiraceae* and *Ruminococcaceae,* which have been associated with stress and social avoidance behavior [64, 65]. Ten genera of the *Lachnospiraceae* family were more abundant in mice^ADHD^ compared to mice^control^; in the latter group, three other *Lachnospiraceae* genera were enriched. Genera of the *Lachnospiraceae* family have been associated with immune responses and are able to control regulatory T cells [66].

Our correlation analysis showed that there was a positive correlation between white matter integrity and the relative abundance of *Holdemania* and *Eubacterium*. These bacteria are both known to be changed in abundances in other disorders: *Holdemania* is increased in individuals with MDD [67–69], and *Eubacterium* is decreased in children with ASD [70]. However, to our knowledge, this is the first study that found significant correlations between these bacteria and brain structural and functional changes. Based on our finding, it would therefore be of interest to investigate this further in the clinical populations of the mentioned disorders.

Changes in the relative abundance of the family *Ruminococcaceae* have been observed in multiple psychiatric and neurodevelopmental diseases including ADHD, ASD, and MDD [71, 72]. Our results show that three genera, *Anaerostipes*, *Roseburia* (both from the *Lachnospiraceae* family), and *Ruminococcaceae UGC-004* (Family *Ruminococcaceae)*, positively correlated with anxiety levels, while *Eubacterium* appeared to be negatively correlated to the severity of anxiety and positively with anxiety reduction. Importantly, these genera were also found to be more abundant in persons with ADHD, compared to controls, as part of our ongoing study of ADHD patients from the NeuroIMAGE study [73] (*n* = 56) and healthy participants (*n* = 49) [74].

This work should be considered in the light of its strengths and limitations. First, we only used six donor samples (three of individuals with ADHD and three of controls) in our study. While this low number of samples may possibly reduce the general distribution of taxonomic groups observable in adult individuals with ADHD, the samples used were carefully selected based only on the disease status (not on behavioral or MRI-based variables in these individuals). To reduce individual differences, samples were pooled before colonization (ADHD patients or controls). Additionally, the choice of collecting the fecal samples from adult male donors was based on the fact that the adult form of ADHD is thought to be the most severe outcome of this disorder [75] and to avoid sex effects and non-controlled hormonal effects, like the use of contraceptives and the menstrual cycle. These hormonal effects are likely to play a role in the shaping of the gut microbial composition, both in humans and mice [76–79], and therefore only male mice were colonized. Besides the hormonal effects, it is possible that unmeasured factors of our donors, e.g., body mass index, also influenced the microbial composition. Though, based on the inclusion criteria of the subjects in the NeuroImage cohort [73], we are confident that the subjects in this study were physically healthy.

Second, we used germ-free mice, housed in isolators, as opposed to antibiotic-treated mice. While this approach limited our behavioral testing options (in terms of equipment), the sterile isolators used in this study provided a contamination-free environment at the time of behavioral testing. Additionally, this allowed us to make sure that the animals were only exposed to the microorganisms from the donor samples, and competition between the native and newly transplanted micro-organisms in germ-free animals was absent, which allows for the study of a defined microbial composition [80]. However, germ-free mice show various developmental and physiological differences when compared to conventionally raised animals. For example, germ-free mice show decreased anxiety and decreased expression of genes that are connected to synaptogenesis [12, 81]. Nevertheless, germ-free mice, housed in isolators, still seem to be the best controlled animal model to study the impact of microbial transplantation [80]. Moreover, by including a group of germ-free mice that received the transplantation from humans without ADHD, a positive control was present. However, it might be interesting to add a third group of germ-free mice that is not colonized (negative control or mock) in future studies, to be able to investigate baseline behavioral (i.e., anxiety) and brain structural and functional indices.

Third, we analyzed the relative microbial composition using 16S rRNA sequencing data. This approach is preferred for comparison across different samples (i.e., different strains of mice), treatments, or timepoints. Additionally, 16S rRNA sequencing techniques are more cost-efficient than metagenomics sequencing; it shows lower signal distortion due to host contamination [82] and has many well-developed analytical tools available [83]. Moreover, we (i) used mock communities in the sequencing experiment in order to check for the “true” bacterial distribution (which validated our taxonomic distribution), (ii) focused on common taxa, and (iii) applied a stringent QC for the statistical analysis to reduce the chance of false positives. However, the choice of sequencing primers (V1-V2 variable region) includes a bias in the sensitivity to identify specific bacterial taxa, for example, the *Bifidobacteria* and *Faecalibacterium* that were observed to differ between ADHD patients and controls [34, 35], could not be measured properly in our samples. Therefore, sequencing the same region of the 16S rRNA gene and the use of similar analysis pipelines is needed in order to properly compare studies in the best possible way.

## Conclusions

In conclusion, bacterial components of the gut microbiota of individuals with ADHD are associated with changes in brain structure and function, as well as behavior in mice. Although we do not suggest that ADHD is caused by changes in microbial composition, the observed changes at the brain level, albeit not specific to ADHD, highlight the relevance of the gut microbiota, potentially through decreased myelination. While further research is needed, our findings might increase our understanding of the etiology of psychiatric disorders and ultimately may open a window of opportunity for the development of potentially novel treatment strategies targeting the microbiota in neurodevelopmental diseases.

## Methods

### Human Participants

Fecal samples were collected from male participants with ADHD (*n* = 3) and age-matched healthy male participants (*n* = 3), with an average age of 22.7 ± 1.1 years. The samples were pooled to prevent transplanting individual microbial compositions [29] and prepared as described (see Additional file 2). The participants were drawn from the follow-up of the NeuroIMAGE study (NeuroIMAGE II) [38], in which participants with ADHD had been diagnosed based on DSM-IV criteria using the Kiddie Schedule for Affective Disorders and Schizophrenia for School-Age Children (K-SADS) [39]. The age of the participants at the time of the K-SADS and feces donation is described in Table 2. Institutional Review Board approval (registration number 2012/542; NL nr.: 41950.091.12) was obtained for the study, and all participants provided written informed consent.

**Table 2:** Characteristics of participants

Variable	ADHD	Control
Men (%)	*N* = 3 (100%)	*N* = 3 (100%)
Age in years at the time of the K-SADS questionnaire (± SEM)	22.0 (1.2)	18.3 (1.5)
Age in years at the time of the feces donation (± SEM)	23.7 (1.2)	20.3 (1.5)
Medication use (% of participants)	Stimulant medication (33.3%)	No

### Experimental design

All animal experiments were carried out in accordance with international European ethical standards (European Directive 2010/63/EU) and were approved by the Veterinary Authority of the Radboud University Medical Center (Radboudumc; permit number: RU-DEC 2015-0077) containing a statistical power analysis to minimize group sizes. All applicable (inter)national and institutional guidelines for the care and use of animals were followed and reported in accordance with the ARRIVE guidelines [40].

**Fig. 5:**
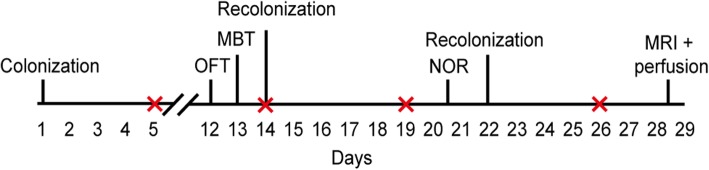
Study design. Germ-free mice were colonized with human ADHD or human control microbiota on day 1 of the experiment. Booster inoculations to recolonize the mice were given on day 14 and 22. Fecal pellets were collected on day 5, 14 (before recolonization), 19, and 26. The open field test (OFT) was performed on day 12, the marble burying test (MBT) on day 13, and the Novel object recognition test (NOR) on day 20 (0.5 h interval) and day 21 (1 h interval). The mice underwent MRI on day 28 or 29, after which all brains were processed for immunohistochemical staining

In total, 28 male, germ-free C57BL/6JOlaHsd mice with an average age of 38 ± 0.5 days were used for this randomized and blinded controlled study. The timeline of the study is illustrated in Fig. 5. Mice were colonized with microbiota from participants with ADHD (“mice^ADHD^”) or healthy participants (“mice^control^”) via oral gavage. Animals were assigned to one of the two BioFlex™ B30 Flexible Film Isolators (Bell Isolation Systems, Livingston, UK), in which the animals were group-housed in standard cages with three or four animals per cage (see Additional file 1: Figure S4). On 27 days post colonization (dpc), mice were transported to the Preclinical Imaging Centre (PRIME) in the central animal facility (CDL) of the Radboud University where the animals were group-housed and scanned with MRI in a random order over the following 2 days. Before the scanning, the mice were housed in Digital Ventilated Cages (Digital Ventilated Cage, Tecniplast S.P.A., Buguggiate (VA) Italy) to study 24/7 activity using a capacitive-based sensor placed non-intrusively under the home cage on the cage rack [41, 42]. To ensure that the gut microbial composition of the mice remained stable throughout the experiment, mice were given booster oral inoculations at 14 and 22 dpc with the same prepared microbiota samples stored at − 80 °C. Fecal pellets were collected weekly between 9 a.m. and 9:30 a.m., snap-frozen, and stored at − 80 °C. One mouse^ADHD^ and one mouse^control^ were euthanized due to complications occurring after the oral gavage on dpc 15 and 22, respectively. All remaining mice underwent MR imaging and were sacrificed directly afterwards using transcardial perfusion fixation. Brains were then collected and immunohistochemistry performed on brain sections (see Additional File 2).

### Microbiota methods and measures

Bacterial DNA was extracted using a repeated bead beating protocol and purified using a customized Maxwell 16 Tissue LEV Total RNA Purification Kit (AS1220; Promega Corporation, Madison, WI, USA). This kit was adapted by the company in order to use it for bacterial DNA extraction (XAS 1220 kit). Amplification of the specific V1-2 region of the 16S rRNA gene was performed. The PCR product was purified using the HighPrep™ PCR kit (MagBio Genomics Inc., Gaithersburg, MD, USA), and libraries with final loading concentrations of 200 ng/μl were prepared for Illumia HiSeq^TM^ sequencing (ATC Biotech AG, Konstanz, Germany), consisting of 46 randomly mixed samples and 2 positive controls (mock communities). The sequenced data was run through the NG-Tax 16S rRNA pipeline [43]. Then as a first control, the composition of the human and mouse samples was compared and plotted in a PCoA figure (see Additional file 1: figure S5 and Additional file 2). After this, the human samples were removed from further analysis to be able to define the specific changes between the mice^ADHD^ and mice^control^. Additionally, due to the microbiome changes after each (re-)colonization, a weighted average was calculated for each animal microbial composition, and quality control was performed in several steps (see Additional file 2). The cleaned data were used to measure within- and between-sample bacterial community diversity. For the within-sample comparison, the following alpha-diversity metrics were used: (1) the number of species present, measured using the observed species richness estimator (R), (2) number of species present and the equitability of each species present as measured by the richness and evenness estimators Shannon-Wiener diversity Index (H’; sensitive to the addition of rare species) and Inverse Simpson diversity index (D2; insensitive to the addition of rare species) [44], and (3) phylogenetic diversity (PD), which takes evolutionary distance of the present bacterial species into account.

Between-sample diversity was measured via the phylogenetic-based assessment of difference in overall bacterial community composition (weighted UniFrac). Linear discriminant analysis effect size (LEfSe) was used to evaluate differences in taxa between the two experimental groups. Genera that differed significantly were selected for correlation analysis to link bacterial changes with behavior and brain parameters.

### Magnetic resonance imaging (MRI)

All MRI measurements were performed using an 11.7-T BioSpec Avance III small animal MR system (Bruker BioSpin, Ettlingen, Germany). Isoflurane (Abbott Animal Health, Abbott Park, IL, USA) was used for anesthesia (induction with 3.5% and maintenance with ~ 1.7% isoflurane in a 1:2 oxygen-air mixture). Imaging parameters can be found in table S1 (Additional file 3: Table S1).

To investigate brain diffusivity, DTI was used as an imaging biomarker for white and gray matter integrity. Fractional anisotropy (FA) is a marker of the degree of myelination and fiber density of white matter (WM), while mean diffusivity (MD) characterizes an inverse measure of the membrane density. Axial diffusivity (AD) and radial diffusivity (RD) describe the parallel and perpendicular directions of diffusivity, respectively [45]. These scalars were derived from the tensor estimation as described (see Additional file 2) [46].

To assess cerebral blood flow (CBF), we acquired MR perfusion data under resting conditions using standardized protocols as described (see Additional file 2) [47]. We evaluated FC patterns using rs-fMRI as described (see Additional file 2) [47].

Hippocampus volumes were calculated from the anatomical T2*weighted images of the MRI scans (see Additional file 3: Table S1 for imaging parameters) using ImageJ (National Institute of Health, Bethesda, MD, USA) (see Additional file 2).

### Behavioral tests

#### Open field test

Mice were allowed to freely explore the square open field (40 × 40 × 25 cm) with white Plexiglas walls for 15 min. We followed a protocol that has been previously described [48].

#### Marble burying test

The MBT was conducted in a standard-sized cage (37 × 19 × 13 cm) preloaded with 3-cm unused sterile bedding and 15 evenly spaced sterilized black glass marbles with a 14-mm diameter. A protocol previously described was used [49].

#### Novel object recognition

This test was performed on dpc 20 (first acquisition day; 30 min delay) and dpc 21 (second acquisition day; 60 min delay) as previously described [47]. Preference for the novel object was expressed as a discrimination index, which is defined as the exploration time for the novel object minus that for the familiar object divided by the total amount of exploration of both objects.

### Statistical analyses

Data were analyzed using the IBM SPSS for Windows 22.0 software (SPSS Inc., Chicago, IL, USA) or R (version 3.2.4). Datasets were analyzed using one-way univariate analysis of variance (normally distributed) or Kruskal-Wallis test (not normally distributed) with Bonferroni post hoc tests, where applicable. Beta-diversity was analyzed using Permanova-S with 10,000 permutations [50]. LEfSe was calculated using the Galaxy Module online (https://huttenhower.sph.harvard.edu/galaxy). The alpha value for the factorial Kruskal-Wallis test among the colonization groups was set to 0.05, and the threshold logarithmic linear discriminant analysis (LDA) score for discriminant features was 2.0. Pearson’s correlation coefficients between microbial composition and key neurobiological findings were calculated (*p* < .01) and plotted using the corrplot package in R. Statistical outliers were removed from the dataset. The number of mice that was considered an outlier per test is described (see Additional file 3: Table S2).

## Supplementary information

**Additional file 1: **Title of data: Supplementary figures. **Figure S1.** Home-cage activity during night and day. **Figure S2.** Novel object recognition test with 1 h interval between the familiarization and test phase. **Figure S3.** Number of marbles buried during the marble burying test. **Figure S4.** Representative photo images of the gnotobiotic isolators that were used in this study. **Figure S5.** Beta-diversity analyses including the human donors.

**Additional file 2: **Title of data*:* Supplemental methods and results. Description: Additional methods and results are available in the supplementary text, including methods of (1) experimental design, (2) preparation of human fecal samples, (3) microbiota analysis, (4) behavioral tests, (5) MRI protocols, (6) immunohistochemistry, and (6) statistical analysis. Additional results are also available including cerebral blood flow, total correlation analyses, and immunohistochemical stainings.

**Additional file 3: **Title of data: Supplementary tables. **Table S1.** Imaging parameters for the anatomical references, rsfMRI, ASL and DTI. **Table S2.** Statistical outliers which were removed from the dataset per test.

## Data Availability

The datasets used and/or analyzed during the current study are available, http://gofile.me/389Ws/xRRVBW2AC.

## References

[1] Ly V, Bottelier M, Hoekstra PJ, Arias Vasquez A, Buitelaar JK, Rommelse NN (2017). Elimination diets’ efficacy and mechanisms in attention deficit hyperactivity disorder and autism spectrum disorder. Eur Child Adolescent Psychiatry.

[2] Rajilic-Stojanovic M, de Vos WM (2014). The first 1000 cultured species of the human gastrointestinal microbiota. FEMS Microbiol Rev.

[3] Borre YE, Moloney RD, Clarke G, Dinan TG, Cryan JF (2014). The impact of microbiota on brain and behavior: mechanisms & therapeutic potential. Adv Exp Med Biol.

[4] Dinan TG, Cryan JF (2017). Microbes, immunity, and behavior: psychoneuroimmunology meets the microbiome. Neuropsychopharmacology.

[5] Petra AI, Panagiotidou S, Hatziagelaki E, Stewart JM, Conti P, Theoharides TC (2015). Gut-Microbiota-brain axis and its effect on neuropsychiatric disorders with suspected immune dysregulation. Clin Ther.

[6] Carabotti M, Scirocco A, Maselli MA, Severi C (2015). The gut-brain axis: interactions between enteric microbiota, central and enteric nervous systems. Ann Gastroenterol.

[7] Kelly JR, Keane VO, Cryan JF, Clarke G, Dinan TG (2019). Mood and microbes: gut to brain communication in depression. Gastroenterol Clin North Am.

[8] Liu P, Peng G, Zhang N, Wang B, Luo B (2019). Crosstalk between the gut microbiota and the brain: an update on neuroimaging findings. Front Neurol.

[9] Hoban AE, Stilling RM, Ryan FJ, Shanahan F, Dinan TG, Claesson MJ, Clarke G, Cryan JF (2016). Regulation of prefrontal cortex myelination by the microbiota. Transl Psychiatry.

[10] Neufeld KM, Kang N, Bienenstock J, Foster JA (2011). Reduced anxiety-like behavior and central neurochemical change in germ-free mice. Neurogastroenterol Motil.

[11] Desbonnet L, Clarke G, Shanahan F, Dinan TG, Cryan JF (2014). Microbiota is essential for social development in the mouse. Mol Psychiatry.

[12] Diaz Heijtz R, Wang S, Anuar F, Qian Y, Bjorkholm B, Samuelsson A, Hibberd ML, Forssberg H, Pettersson S (2011). Normal gut microbiota modulates brain development and behavior. Proc Natl Acad Sci U S A.

[13] Ogbonnaya ES, Clarke G, Shanahan F, Dinan TG, Cryan JF, O'Leary OF (2015). Adult hippocampal neurogenesis is regulated by the microbiome. Biological Psychiatry.

[14] Luczynski P, Whelan SO, O'Sullivan C, Clarke G, Shanahan F, Dinan TG, Cryan JF (2016). Adult microbiota-deficient mice have distinct dendritic morphological changes: differential effects in the amygdala and hippocampus. Eur J Neurosci.

[15] Al-Amin M, Zinchenko A, Geyer T (2018). Hippocampal subfield volume changes in subtypes of attention deficit hyperactivity disorder. Brain Res.

[16] Hoogman M, Bralten J, Hibar DP, Mennes M, Zwiers MP, Schweren LSJ, van Hulzen KJE, Medland SE, Shumskaya E, Jahanshad N (2017). Subcortical brain volume differences in participants with attention deficit hyperactivity disorder in children and adults: a cross-sectional mega-analysis. Lancet Psychiatry.

[17] Plessen KJ, Bansal R, Zhu H, Whiteman R, Amat J, Quackenbush GA, Martin L, Durkin K, Blair C, Royal J (2006). Hippocampus and amygdala morphology in attention-deficit/hyperactivity disorder. Arch Gen Psychiatry.

[18] Ong IM, Gonzalez JG, McIlwain SJ, Sawin EA, Schoen AJ, Adluru N, Alexander AL, Yu JJ (2018). Gut microbiome populations are associated with structure-specific changes in white matter architecture. Transl Psychiatry.

[19] Cryan JF, O'Riordan KJ, Cowan CSM, Sandhu KV, Bastiaanssen TFS, Boehme M, Codagnone MG, Cussotto S, Fulling C, Golubeva AV (2019). The microbiota-gut-brain axis. Physiological reviews.

[20] Borre YE, O'Keeffe GW, Clarke G, Stanton C, Dinan TG, Cryan JF (2014). Microbiota and neurodevelopmental windows: implications for brain disorders. Trends Mol Med.

[21] Bercik P, Denou E, Collins J, Jackson W, Lu J, Jury J, Deng Y, Blennerhassett P, Macri J, McCoy KD (2011). The intestinal microbiota affect central levels of brain-derived neurotropic factor and behavior in mice. Gastroenterology.

[22] Degroote S, Hunting DJ, Baccarelli AA, Takser L (2016). Maternal gut and fetal brain connection: Increased anxiety and reduced social interactions in Wistar rat offspring following peri-conceptional antibiotic exposure. Progress Neuro-psychopharmacol Biol Psychiatry.

[23] Arentsen T, Raith H, Qian Y, Forssberg H, Diaz Heijtz R (2015). Host microbiota modulates development of social preference in mice. Microbial Ecol Health Dis.

[24] Bruch JD (2016). Intestinal infection associated with future onset of an anxiety disorder: results of a nationally representative study. Brain Behav Immun.

[25] Zijlmans MAC, Korpela K, Riksen-Walraven JM, de Vos WM, de Weerth C (2015). Maternal prenatal stress is associated with the infant intestinal microbiota. Psychoneuroendocrinology.

[26] Allen AP, Hutch W, Borre YE, Kennedy PJ, Temko A, Boylan G, Murphy E, Cryan JF, Dinan TG, Clarke G (2016). Bifidobacterium longum 1714 as a translational psychobiotic: modulation of stress, electrophysiology and neurocognition in healthy volunteers. Transl Psychiatry.

[27] Pinto-Sanchez MI, Hall GB, Ghajar K, Nardelli A, Bolino C, Lau JT, Martin F-P, Cominetti O, Welsh C, Rieder A (2017). Probiotic Bifidobacterium longum NCC3001 reduces depression scores and alters brain activity: a pilot study in patients with irritable bowel syndrome. Gastroenterology.

[28] Cryan JF, Dinan TG (2015). More than a gut feeling: the microbiota regulates neurodevelopment and behavior. Neuropsychopharmacology.

[29] Fond G, Boukouaci W, Chevalier G, Regnault A, Eberl G, Hamdani N, Dickerson F, Macgregor A, Boyer L, Dargel A (2015). The “psychomicrobiotic”: targeting microbiota in major psychiatric disorders: a systematic review. Pathol Biol.

[30] Liu F, Li J, Wu F, Zheng H, Peng Q, Zhou H (2019). Altered composition and function of intestinal microbiota in autism spectrum disorders: a systematic review. Transl Psychiatry.

[31] Cenit MC, Nuevo IC, Codoner-Franch P, Dinan TG, Sanz Y (2017). Gut microbiota and attention deficit hyperactivity disorder: new perspectives for a challenging condition. Eur Child Adolesc Psychiatry.

[32] Srikantha P, Mohajeri MH. The possible role of the microbiota-gut-brain-axis in autism spectrum disorder. *Int J Mol Sci*. 2019:20. 10.3390/ijms20092115 PMC6539237 31035684

[33] Kelly JR, Borre Y, OB C, Patterson E, El Aidy S, Deane J, Kennedy PJ, Beers S, Scott K, Moloney G (2016). Transferring the blues: depression-associated gut microbiota induces neurobehavioural changes in the rat. J Psychiatr Res.

[34] Aarts E, Ederveen THA, Naaijen J, Zwiers MP, Boekhorst J, Timmerman HM, Smeekens SP, Netea MG, Buitelaar JK, Franke B (2017). Gut microbiome in ADHD and its relation to neural reward anticipation. PLoS One.

[35] Jiang HY, Zhou YY, Zhou GL, Li YC, Yuan J, Li XH, Ruan B (2018). Gut microbiota profiles in treatment-naive children with attention deficit hyperactivity disorder. Behav Brain Res.

[36] Nigg JT, Lewis K, Edinger T, Falk M (2012). Meta-analysis of attention-deficit/hyperactivity disorder or attention-deficit/hyperactivity disorder symptoms, restriction diet, and synthetic food color additives. J Am Acad Child Adolesc Psychiatry.

[37] Sonuga-Barke EJS, Brandeis D, Cortese S, Daley D, Ferrin M, Holtmann M, Stevenson J, Danckaerts M, van der Oord S, Dopfner M (2013). Nonpharmacological interventions for ADHD: systematic review and meta-analyses of randomized controlled trials of dietary and psychological treatments. Am J Psychiatry.

[38] Pelsser LM, Frankena K, Toorman J, Rodrigues Pereira R (2017). Diet and ADHD, reviewing the evidence: a systematic review of meta-analyses of double-blind placebo-controlled trials evaluating the efficacy of diet interventions on the behavior of children with ADHD. PloS One.

[39] Stevenson J, Buitelaar J, Cortese S, Ferrin M, Konofal E, Lecendreux M, Simonoff E, Wong ICK, Sonuga-Barke E (2014). Research review: the role of diet in the treatment of attention-deficit/hyperactivity disorder--an appraisal of the evidence on efficacy and recommendations on the design of future studies. J Child Psychol Psychiatry Allied Disciplines.

[40] Ramirez PL, Barnhill K, Gutierrez A, Schutte C, Hewitson L (2013). Improvements in behavioral symptoms following antibiotic therapy in a 14-year-old male with autism. Case Rep Psychiatry.

[41] Sandhu KV, Sherwin E, Schellekens H, Stanton C, Dinan TG, Cryan JF (2017). Feeding the microbiota-gut-brain axis: diet, microbiome, and neuropsychiatry. Transl Res.

[42] Alexander AL, Hurley SA, Samsonov AA, Adluru N, Hosseinbor AP, Mossahebi P, Tromp DPM, Zakszewski E, Field AS (2011). Characterization of cerebral white matter properties using quantitative magnetic resonance imaging stains. Brain Connectivity.

[43] Taylor GT, Lerch S, Chourbaji S (2017). Marble burying as compulsive behaviors in male and female mice. Acta Neurobiol Exp.

[44] Hueston CM, Cryan JF, Nolan YM. Stress and adolescent hippocampal neurogenesis: diet and exercise as cognitive modulators. *Transl Psychiatry*. 2017;7. 10.1038/tp.2017.48 PMC5416690 28375209

[45] Jacka FN, Cherbuin N, Anstey KJ, Sachdev P, Butterworth P (2015). Western diet is associated with a smaller hippocampus: a longitudinal investigation. BMC Med.

[46] Mohle L, Mattei D, Heimesaat MM, Bereswill S, Fischer A, Alutis M, French T, Hambardzumyan D, Matzinger P, Dunay IR, Wolf SA (2016). Ly6C(hi) monocytes provide a link between antibiotic-induced changes in gut microbiota and adult hippocampal neurogenesis. Cell Rep.

[47] Perlov E, Philipsen A (2008). Tebartz van Elst L, Ebert D, Henning J, Maier S, Bubl E, Hesslinger B: Hippocampus and amygdala morphology in adults with attention-deficit hyperactivity disorder. J Psychiatry Neurosci.

[48] Zuo C, Wang D, Tao F, Wang Y. Changes in the development of subcortical structures in autism spectrum disorder. *Neuroreport*. 2019. 10.1097/WNR.0000000000001300 31464839

[49] Li Y, Shen M, Stockton ME, Zhao X. Hippocampal deficits in neurodevelopmental disorders. *Neurobiol Learning Memory*. 2018. 10.1016/j.nlm.2018.10.001 PMC6461531 30321651

[50] Kang E, Wen Z, Song H, Christian KM, Ming G-L. Adult neurogenesis and psychiatric disorders. *Cold Spring Harbor Perspectives Biol*. 2016;8. 10.1101/cshperspect.a019026 PMC5008067 26801682

[51] Chen L, Hu X, Ouyang L, He N, Liao Y, Liu Q, Zhou M, Wu M, Huang X, Gong Q (2016). A systematic review and meta-analysis of tract-based spatial statistics studies regarding attention-deficit/hyperactivity disorder. Neurosci Biobehav Rev.

[52] Onnink AM, Zwiers MP, Hoogman M, Mostert JC, Dammers J, Kan CC, Vasquez AA, Schene AH, Buitelaar J, Franke B (2015). Deviant white matter structure in adults with attention-deficit/hyperactivity disorder points to aberrant myelination and affects neuropsychological performance. Prog Neuropsychopharmacol Biol Psychiatry.

[53] Fraser JA, Newman NJ, Biousse V (2011). Disorders of the optic tract, radiation, and occipital lobe. Handbook Clin Neurol.

[54] Pastura G, Doering T, Gasparetto EL, Mattos P, Araujo AP (2016). Exploratory analysis of diffusion tensor imaging in children with attention deficit hyperactivity disorder: evidence of abnormal white matter structure. Atten Defic Hyperact Disord.

[55] Safadi Z, Grisot G, Jbabdi S, Behrens TE, Heilbronner SR, McLaughlin NCR, Mandeville J, Versace A, Phillips ML, Lehman JF (2018). Functional segmentation of the anterior limb of the internal capsule: linking white matter abnormalities to specific connections. J Neurosci.

[56] Emos MC, Agarwal S: Neuroanatomy, internal capsule. In *StatPearls.* Treasure Island (FL); 2019. 31194338

[57] Archer DB, Kang N, Misra G, Marble S, Patten C, Coombes SA (2018). Visual feedback alters force control and functional activity in the visuomotor network after stroke. NeuroImage Clin.

[58] Kurdziel LBF, Dempsey K, Zahara M, Valera E, Spencer RMC (2015). Impaired visuomotor adaptation in adults with ADHD. Exp Brain Res.

[59] Bansal S, Murthy KG, Fitzgerald J, Schwartz BL, Joiner WM (2019). Reduced transfer of visuomotor adaptation is associated with aberrant sense of agency in schizophrenia. Neuroscience.

[60] van der Meer D, Hoekstra PJ, van Rooij D, Winkler AM, van Ewijk H, Heslenfeld DJ, Oosterlaan J, Faraone SV, Franke B, Buitelaar JK, Hartman CA. Anxiety modulates the relation between attention-deficit/hyperactivity disorder severity and working memory-related brain activity. *World J Biol Psychiatry*. 2017:1–11. 10.1080/15622975.2017.1287952 PMC5581282 28635543

[61] Reale L, Bartoli B, Cartabia M, Zanetti M, Costantino MA, Canevini MP, Termine C, Bonati M, Lombardy AG, Conte S (2017). Comorbidity prevalence and treatment outcome in children and adolescents with ADHD. Eur Child Adolesc Psychiatry.

[62] Malan-Muller S, Valles-Colomer M, Raes J, Lowry CA, Seedat S, Hemmings SMJ (2018). The gut microbiome and mental health: implications for anxiety- and trauma-related disorders. Omics.

[63] Ma B, Liang J, Dai M, Wang J, Luo J, Zhang Z, Jing J (2019). Altered gut microbiota in chinese children with autism spectrum disorders. Front Cell Infection Microbiol.

[64] Gacias M, Gaspari S, Santos P-MG, Tamburini S, Andrade M, Zhang F, Shen N, Tolstikov V, Kiebish MA, Dupree JL, et al. Microbiota-driven transcriptional changes in prefrontal cortex override genetic differences in social behavior. *eLife*. 2016;5. 10.7554/eLife.13442 PMC4880443 27097105

[65] Li S, Wang Z, Yang Y, Yang S, Yao C, Liu K, Cui S, Zou Q, Sun H, Guo G (2017). Lachnospiraceae shift in the microbial community of mice faecal sample effects on water immersion restraint stress. AMB Express.

[66] Atarashi K, Tanoue T, Oshima K, Suda W, Nagano Y, Nishikawa H, Fukuda S, Saito T, Narushima S, Hase K (2013). Treg induction by a rationally selected mixture of Clostridia strains from the human microbiota. Nature.

[67] Chung Y-CE, Chen H-C, Chou H-CL, Chen IM, Lee M-S, Chuang L-C, Liu Y-W, Lu M-L, Chen C-H, Wu C-S (2019). Exploration of microbiota targets for major depressive disorder and mood related traits. J Psychiatric Res.

[68] Chen Y-H, Bai J, Wu D, Yu S-F, Qiang X-L, Bai H, Wang H-N, Peng Z-W (2019). Association between fecal microbiota and generalized anxiety disorder: severity and early treatment response. J Affective Disord.

[69] Hechler C, Borewicz K, Beijers R, Saccenti E, Riksen-Walraven M, Smidt H, de Weerth C (2019). Association between psychosocial stress and fecal microbiota in pregnant women. Sci Rep.

[70] Liu S, Li E, Sun Z, Fu D, Duan G, Jiang M, Yu Y, Mei L, Yang P, Tang Y, Zheng P (2019). Altered gut microbiota and short chain fatty acids in Chinese children with autism spectrum disorder. Sci Rep.

[71] Hsiao EY, McBride SW, Hsien S, Sharon G, Hyde ER, McCue T, Codelli JA, Chow J, Reisman SE, Petrosino JF (2013). Microbiota modulate behavioral and physiological abnormalities associated with neurodevelopmental disorders. Cell.

[72] Jiang H, Ling Z, Zhang Y, Mao H, Ma Z, Yin Y, Wang W, Tang W, Tan Z, Shi J (2015). Altered fecal microbiota composition in patients with major depressive disorder. Brain Behav Immun.

[73] von Rhein D, Mennes M, van Ewijk H, Groenman AP, Zwiers MP, Oosterlaan J, Heslenfeld D, Franke B, Hoekstra PJ, Faraone SV (2015). The NeuroIMAGE study: a prospective phenotypic, cognitive, genetic and MRI study in children with attention-deficit/hyperactivity disorder. Design and descriptives. Eur Child Adolesc Psychiatry.

[74] Szopinska-Tokov J, Dam S, Naaijen J, Konstanti P, Rommelse N, Belzer C, Buitelaar J, Franke B, Aarts E, Arias Vasquez A: Investigating the Gut Microbiota Composition of Individuals with Attention-Deficit/Hyperactivity Disorder and Association with Symptoms. Microorganisms 2020;8:406. 10.3390/microorganisms8030406. 10.3390/microorganisms8030406 PMC7143990 32183143

[75] Bonvicini C, Faraone SV, Scassellati C (2016). Attention-deficit hyperactivity disorder in adults: a systematic review and meta-analysis of genetic, pharmacogenetic and biochemical studies. Mol Psychiatry.

[76] Yurkovetskiy L, Burrows M, Khan AA, Graham L, Volchkov P, Becker L, Antonopoulos D, Umesaki Y, Chervonsky AV (2013). Gender bias in autoimmunity is influenced by microbiota. Immunity.

[77] Markle JGM, Frank DN, Mortin-Toth S, Robertson CE, Feazel LM, Rolle-Kampczyk U, von Bergen M, McCoy KD, Macpherson AJ, Danska JS (2013). Sex differences in the gut microbiome drive hormone-dependent regulation of autoimmunity. Science (New York, N Y ).

[78] Chen KL, Madak-Erdogan Z (2016). Estrogen and microbiota crosstalk: should we pay attention?. Trends Endocrinol Metabol.

[79] Elderman M, Hugenholtz F, Belzer C, Boekschoten M, van Beek A, de Haan B, Savelkoul H, de Vos P, Faas M (2018). Sex and strain dependent differences in mucosal immunology and microbiota composition in mice. Biol Sex Diff.

[80] Lundberg R, Toft MF, August B, Hansen AK, Hansen CH (2016). Antibiotic-treated versus germ-free rodents for microbiota transplantation studies. Gut Microbes.

[81] Clarke G, Grenham S, Scully P, Fitzgerald P, Moloney RD, Shanahan F, Dinan TG, Cryan JF (2013). The microbiome-gut-brain axis during early life regulates the hippocampal serotonergic system in a sex-dependent manner. Mol Psychiatry.

[82] Hilton SK, Castro-Nallar E, Perez-Losada M, Toma I, McCaffrey TA, Hoffman EP, Siegel MO, Simon GL, Johnson WE, Crandall KA (2016). Metataxonomic and metagenomic approaches vs. culture-based techniques for clinical pathology. Front Microbiol.

[83] Poretsky R, Rodriguez RL, Luo C, Tsementzi D, Konstantinidis KT (2014). Strengths and limitations of 16S rRNA gene amplicon sequencing in revealing temporal microbial community dynamics. PLoS One.

