# Supplementary material for: Gut microbiota from persons with attention-deficit/hyperactivity disorder affects the brain in mice
Source: Microbiome. 2020 Apr 1;8:44. doi: 10.1186/s40168-020-00816-x (PMC7114819; doi:10.1186/s40168-020-00816-x)
Supplement: Supplementary file 2 — Additional file 1: Title of data: Supplementary figures. Figure S1. Home-cage activity during night and day. Figure S2. Novel object recognition test with 1 h interval between the familiarization and test phase. Figure S3. Number of marbles buried during the marble burying test. Figure S4. Representative photo images of the gnotobiotic isolators that were used in this study. Figure S5. Beta-diversity analyses including the human donors. [file 40168_2020_816_MOESM1_ESM.docx]

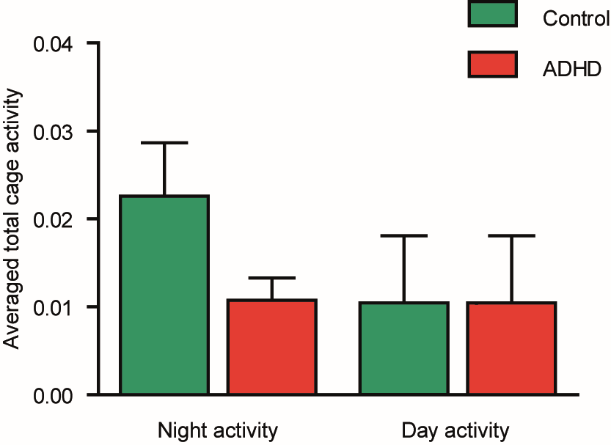


**Figure S1. Home-cage activity.** Averaged total cage activity during the night (left) and day (right). Activity was corrected for the number of mice per cage (4 cages in total: 2 cages with mice^ADHD^ and 2 cages with mice^control^).


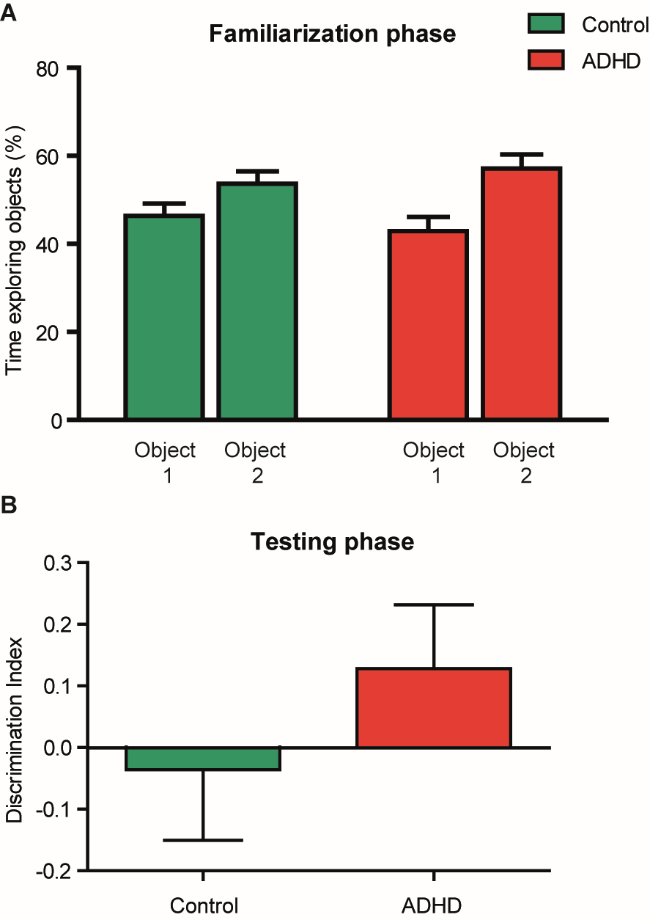


**Figure S2. Novel object recognition test with 1h interval between the familiarization and test phase. (A)** During the familiarization phase of the NOR test mice^control^ (green bars) and mice^ADHD^ (red bars) explored both identical objects equally. **(B)** The discrimination index did not differ between mice^control^ and mice^ADHD^. N=14 mice^control^ and N=13 mice^ADHD^


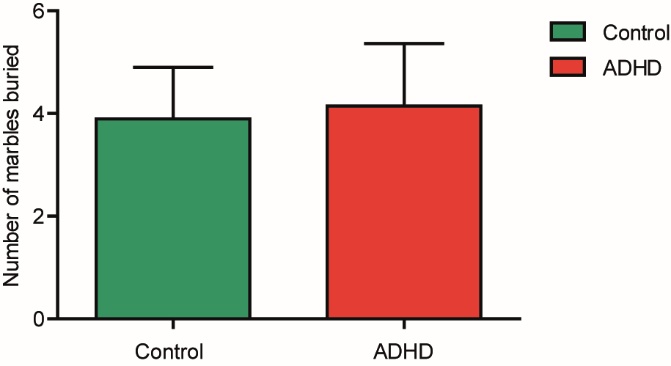


**Figure S3. Number of marbles buried in the MBT.** The number of marbles with at least two-thirds of its size covered by bedding was counted by three evaluators. N=14 per group


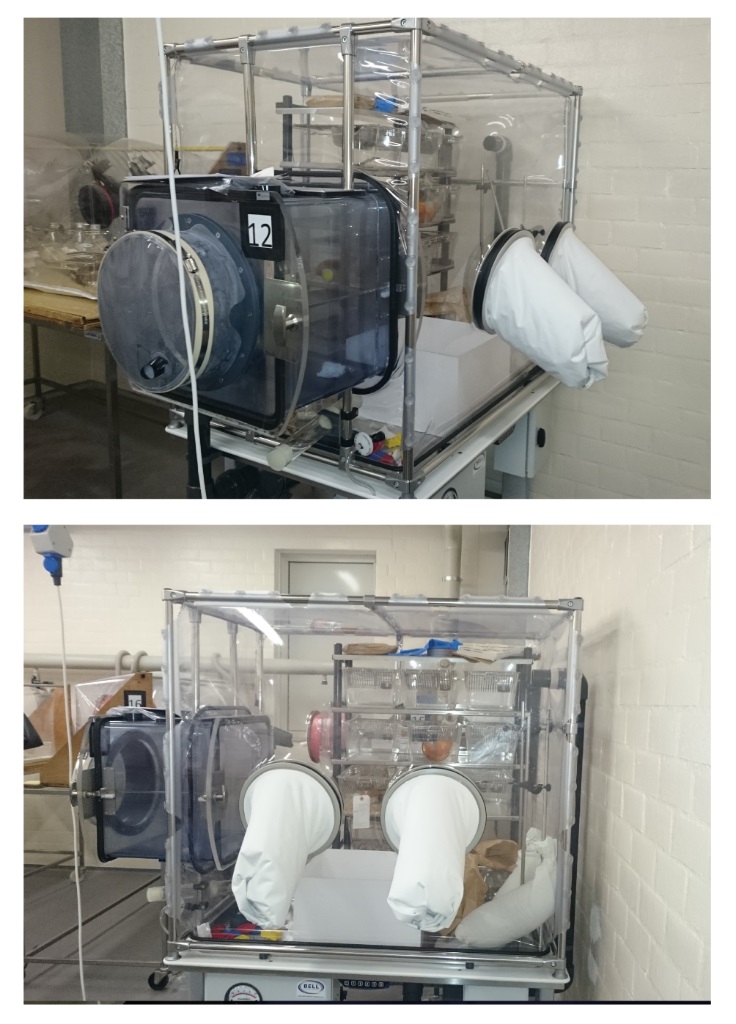


**Figure S4.** Representative photo images of the gnotobiotic isolators that were used in this study.


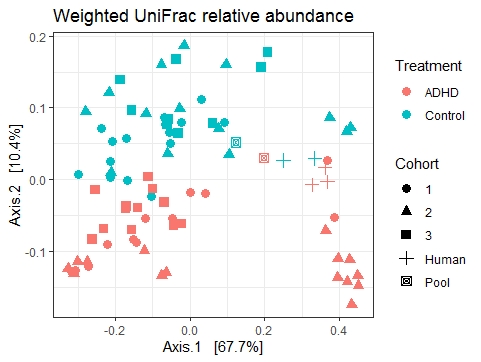


**Figure S5.** **Beta-diversity analyses including the human donors**. Principal coordinate analysis (PCoA) plot of weighted UniFrac distances in which the three human donors per group (ADHD patients or controls) and each collected mice sample are plotted. The plot shows separation in microbial composition between mice^ADHD^ and mice^control^ and clustering of the human donors.
