# Supplementary material for: Gut microbiota from persons with attention-deficit/hyperactivity disorder affects the brain in mice
Source: Microbiome. 2020 Apr 1;8:44. doi: 10.1186/s40168-020-00816-x (PMC7114819; doi:10.1186/s40168-020-00816-x)
Supplement: Supplementary file 3 — Additional file 2: Title of data: Supplemental methods and results. Description: Additional methods and results are available in the supplementary text, including methods of (1) experimental design, (2) preparation of human fecal samples, (3) microbiota analysis, (4) behavioral tests, (5) MRI protocols, (6) immunohistochemistry, and (6) statistical analysis. Additional results are also available including cerebral blood flow, total correlation analyses, and immunohistochemical stainings. [file 40168_2020_816_MOESM2_ESM.docx]

**Additional file 1**

**Detailed methods**

**Mice**

C57BL/6JOlaHsd wild-type mice originated from Harlan Laboratories Inc. (now Envigo, Horst, the Netherlands) and made germ-free by hysterectomy rederivation. A germ-free colony was then established at the Central Animal Laboratory (CDL; Radboudumc Nijmegen, the Netherlands). The mice were tested in three separate batches (Batch A: mice^control^ n=6, mice^ADHD^ n=5; Batch B: mice^control^ n=5, mice^ADHD^ n=5; Batch C: mice^control^ n=3, mice^ADHD^ n=4; total mice^control^: n=14, total mice^ADHD^ n=14). Animals were housed in BioFlex™ B30 Flexible Film Isolators (Bell Isolation Systems, Livingston, UK),under standard laboratory conditions (temperature: 21°C, relative humidity: 50%-60%, room light: 60 lux, 12-hour light/dark cycle: 7 a.m.-7 p.m.). All behavioral experiments were performed inside the gnotobiotic isolators during the light phase, between 9:30 a.m. and 12:30 p.m. Care was taken to prevent cage effects by a number of measures: (1) animals were randomly assigned to the two treatment groups. Littermates were distributed over treatments and cages before the start of the experiment. Mice were tested in a random order; (2) treatment groups were concealed to everyone involved; (3) All mice were handled equally, and all cages were cleaned once a week; (4) animals were housed in standard cages inside the isolator. The location of these cages inside the isolator was switched two times per week. The location of the isolators also switched twice during the experiment; (5) Both isolators were placed in the same room with no other animals present. Care was taken to limit the differences in environmental conditions (e.g., light intensity, shadows, sounds, ventilation and temperature). Mice had *ad libitum* access to autoclaved mouse chow (V1534-300; Ssniff Spezialdiäten GmbH, Soest, the Netherlands) and sterilized demineralized water. Animals were group-housed in standard cages with three or four animals per cage. On dpc 27, mice were transported to the Preclinical Imaging Centre (PRIME) in the central animal facility (CDL) of the Radboud University where the animals were group-housed for one to two days awaiting MRI scanning. The mice were housed in Digital Ventilated Cages (Digital Ventilated Cage, Tecniplast S.P.A., Buguggiate (VA) Italy) to study 24/7 activity using a capacitive-based sensor placed non-intrusively under the home cage on the cage rack [1, 2].

**Preparation of human fecal samples for colonization**

Human fecal samples were kept on ice immediately after receiving and stored at -80°C until processing. The fecal samples were prepared for colonization by pooling equal amounts of fecal material. The pooled samples were diluted with sterile Phosphate Buffered Saline (0.1M PBS; pH 7.3) to make a 10% (w/v) dilution. The pooled samples were vortexed at room temperature for 5 minutes after which the suspension was allowed to settle by gravity for 5 minutes on ice. Finally, the supernatant was divided into 200 µL aliquots and stored at -80°C.

**PCR amplification and Illumina sequencing**

Bacterial DNA with a concentration of 20 ng/µl was used for the amplification of the specific V1-2 region of the 16S rRNA gene using previously reported primers of this region: 27F-DegS (5’GTTYGATYMTGGCTCAG) – 338RI-II (5’ GCWGCC[A/T]CCCGTAGG[T/A]GT) [3-6]. The PCR protocol contained the following steps: first, a denaturation step at 98°C for 30 seconds, then 25 cycles with the following conditions: 98°C for 10 seconds, 56°C for 20 seconds and 72°C for 20 seconds. Finally, the product was kept at 72°C for 10 minutes and then cooled down to 4°C. The size of the PCR product was checked using SYBR® Green Supermix on 1% agarose gel. Second PCR consists of five cycles, with the same condition as described above, to add unique labels (barcodes) to the fragments of each sample by forward and revered primers to be able to identify the bacterial community per sample after sequencing. The PCR product was checked, as in the first PCR, after the PCR product was purified using the HighPrep™ PCR kit (CleanPCR). For each purified sample the DNA concentration was measured using Qubit® 2.0 fluorometer and libraries were prepared. Each library consists of randomly mixed 46 samples and 2 positive controls (mock communities). Final loading concentration of libraries was targeted at 200ng/ µl. The library was sent for Illumia HiSeq^TM^ sequencing to GATC Biotech AG (Konstanz, Germany).

**Analysis of 16S rRNA amplicons and quality control**

The sequenced data was received from GATC Biotech AG (Konstanz, Germany) and was put through NG-Tax 16S rRNA pipeline at Wageningen University and Research (WUR, Wageningen, NL) [7]. The NG-Tax pipeline is a tool to identify the taxonomy of samples based on 16S rRNA sequences and consists of three core elements: barcode-primer filtering, OTU clustering based on having ≥98.5% sequence similarities and taxonomic assignment using Levenshtein distance clustering algorithm and SILVA database. On the output file of NG-Tax (BIOM-file) several quality control (QC) steps were performed. The OTU table was first filtered for OTUs present in less than 10% of the samples, followed by eliminating OTU sequences that represented less than 1% of total reads. Samples which contained less than 10% of OTUs were removed. Finally, a weighted average of bacterial abundances was created from the different collection time points to correct for the effect of time and recolonization.

**Diversity metrics**

To compare within-sample bacterial community diversity, multiple alpha-diversity metrics were used: 1. species richness estimator (R) counting the observed unique OTUs in each sample, 2. richness and evenness estimators Shannon-Wiener diversity Index (H’) and Inverse Simpson diversity index (D_2_) [8] 3. phylogenetic richness estimator takes into consideration the phylogeny of microbes to estimate diversity across a tree (phylogenetic diversity (PD)). To compare between-sample diversity, a phylogenetic-based assessment of difference in overall bacterial community composition was used called weighted UniFrac and results are presented by a Principal coordinate analysis (PCoA) plot.

**Behavioral tests**

Open Field (OFT): Mice were placed in the center of the square open field (40 x 40 x 25 cm) with white Plexiglas walls, and allowed to freely explore the area for 15 minutes. The trials were videotaped, and exploration activity in the center (20 x 20 cm) and corners (10 x10 cm) was automatically scored using Ethovision XT10.1 (Noldus, Wageningen, The Netherlands). In addition, the durations (in seconds) and frequencies of sitting, rearing, jumping, walking, grooming and leaning behavior, were manually scored and analyzed using the same program. Between trials, the open field arena was thoroughly cleaned with sterilized water and 70% Ethanol.

Marble burying test (MBT): Mice were placed in a clean standard-sized cage preloaded with 3 cm unused bedding and 15 evenly spaced sterilized black glass marbles with a 14 mm diameter. Mice were allowed to explore the cage for 30 minutes, after which they were removed from the cage and the cage was photographed. The number of marbles with at least two-thirds of its size covered by bedding was counted by three evaluators and analyzed using IBM SPSS for Windows 22.0 software (SPSS Inc., Chicago, IL, USA). Between trials, marbles and cages were cleaned with 70% Ethanol.

Novel Object Recognition Test (NORT): The object recognition test (NOR) was performed on dpc 20 (first acquisition day; 30 min delay) and dpc 21 (second acquisition day; 60 min delay) as previously described (see Additional file 1) [9]. The familiarization and test phase were recorded and analyzed using Ethovision XT10.1 (Noldus, Wageningen, the Netherlands). Exploration of an object was defined as pointing the nose to the object at a distance of <1 cm and/or touching it with the nose. Preference for the novel object was expressed in discrimination index, which is defined as the exploration time for the novel object minus the familiar object divided by the total amount of exploration of both objects. The objects used were similar in size, but dissimilar in color, shape and texture. Mice could not move the objects nor could climb over the objects. The objects and arena were thoroughly cleaned with sterilized water and 70% ethanol after each trial to remove odors.

**Magnetic Resonance Imaging (MRI)**

Resting-state fMRI (rsfMRI) acquisition was performed to assess functional connectivity (FC) between specific regions of interest (ROI) that support multiple cognitive and motor processes: dorsal hippocampus, ventral hippocampus, auditory cortex, motor cortex, somatosensory cortex and visual cortex. To minimize the impact of echo planar imaging-related artifacts, which are more prominent in areas of different tissues interface (e.g., near the skull or near the ear canals), we selected all cortical ROIs 1–2 away from the edge of the cortex. FC group comparison between ROIs was calculated from the blood oxygen level dependent (BOLD) time series using total and partial correlation analyses as previously described [10]. The direct connectivity between two ROIs is emphasized by the partial correlations, while the temporal BOLD signal from the other ROIs is regressed.

To measure cerebral blood flow (CBF) under resting conditions, we used arterial spin labeling (ASL) with flow-sensitive alternating inversion recovery (FAIR) [11, 12]. Regional perfusion was measured in specific ROIs: entire brain, cerebral cortex, hippocampus, thalamus, motor cortex, caudate putamen and bed nucleus (bregma between -1.46 and -2.30) [13]. Regional CBF was calculated and analyzed using the same protocol as previously described [14].

Diffusion of water was measured by acquiring 22 axial slices covering the whole brain following a protocol described earlier [10, 15]. The scalars fractional anisotropy (FA), mean water diffusivity (MD), axial (longitudinal) diffusivity (AD) and radial diffusivity (RD) were derived from the tensor estimation as described elsewhere [16]. These scalars were measured in several white matter (WM) and gray matter (GM) areas, manually selected according to the atlas of Franklin and Paxinos [13]. The selected ROIs were the fornix, corpus callosum, and regions in the left and right hemisphere: auditory cortex, caudate putamen, hippocampus, motor cortex, somatosensory cortex, visual cortex, corpus callosum splenium, external capsule, forceps minor, internal capsule and optic tract.

Hippocampus volumes were calculated from MRI scans using ImageJ (National Institute of Health, Bethesda, Maryland, USA). The left and right hippocampus were manually outlined on all anatomical reference images containing the hippocampus. The area size per slice was then multiplied by the slice thickness (0.5 cm) and the values per hippocampus were combined to calculate the volume of each hippocampus.

**Immunohistochemistry**

Mice were sacrificed by transcardial perfusion fixation with 0.1 M phosphate buffered saline (PBS, pH=7.3) followed by 4% paraformaldehyde in 0.1 M PBS at room temperature. Brains were removed, immersion-fixated overnight at 4°C in 4% paraformaldehyde in 0.1 M PBS, and subsequently stored at 4°C in 0.1 M PBS with 0.01% sodium azide before sectioning and immunohistochemical stainings. For immunostaining of the paraffin-embedded brains, 5 µm thick coronal sections were cut (Bregma -0.86 to -1.94). The sections were deparaffinized and stained in one session to minimize variations in staining intensity.

To visualize postsynaptic density protein 95 (PSD95), polyclonal rabbit anti-PSD95 (1:1000; ab18258; Abcam Inc., Cambridge, UK) was used as a primary antibody to analyze the postsynaptic density. The secondary antibody was donkey anti-rabbit biotin (1:200; Jackson ImmunoResearch, West Grove, PA, USA). PSD-95 staining was analyzed using a Zeiss Axioskop microscope equipped with hardware and software of Microbrightfield (Williston, VT, USA). Relevant brain regions were based on the mouse brain atlas of Paxinos and Franklin [13] and quantified in four regions: left and right motor cortex, and left and right caudate putamen (bregma 0.14-0.26). The relevant regions were digitized at 40x magnification using Stereo Investigator software (Microbrightfield, Williston, VT, USA). The staining was quantified using Image J (National Institute of Health, Bethesda, Mayland, USA). The contrast was manually enhanced, following the same procedure for all digitized images, and the amount of tissue stained was measured with a threshold-based approach.

To visualize doublecortin, polyclonal goat anti-DCX (1:200; Santa Cruz Biotechnology Inc., Santa Cruz, CA, USA) was used as a primary antibody to assess neurogenesis. As secondary antibody donkey anti-rabbit biotin was used (1:200; Jackson ImmunoResearch, West Grove, PA, USA). Brain sections (Bregma: -1.94) were preselected for quantification according to the atlas of Franklin and Paxinos [13]. Quantification was performed in the hippocampus, on images at a 5x magnification using an Axio Imager A2 (Zeiss Germany).

**Statistical analysis**

Data were analyzed using IBM SPSS for Windows 22.0 software (SPSS Inc., Chicago, IL, USA) or R (version 3.2.4) using the following packages: Microbiome, Phyloseq, microbiomeutilities, ggpubr, DT, data.table, dplyr, Vegan, and Picante. Normal distribution was evaluated by Shapiro-Wilk test and evaluation of the Q-Q plots. In the case of significant (p<.05) deviations from normal distribution, non-parametric Kruskall Wallis test was used. Alpha diversity was visualized using Graphpad Prism version 5.00 for Windows. For the between samples comparison Permanova-S was used with 10000 permutations [17]. A Linear discriminant analysis effect size (LEfSe) was calculated using the Galaxy Module online (https://huttenhower.sph.harvard.edu/galaxy). In which the alpha value for the factorial Kruskal-Wallis test among the colonization groups was set to 0.05 and the threshold logarithmic linear discriminant analysis (LDA) score for discriminant features was 2.0. Pearson’s Correlation coefficients between microbial composition and key pathological findings (i.e., anxiety and DTI measures of the hippocampus and internal capsule) were calculated and plotted using the corrplot package in R. Statistical significance was set at *p*<0.05 (*), *p*<0.01 (**), and *p*<0.001 (***). Relevant statistics are given for each statistical analysis.

**Supplementary Results**

*Analysis of human and individual mice samples*

Principal coordinate analysis (PCoA) plot of weighted UniFrac distances was created to visualize the microbial composition (beta-diversity) of the individual samples of the mice, the original human donated fecal samples and the pooled samples. These latter samples are a mix of the three human samples per group (ADHD or control) that were transplanted into the mice. By pooling the samples, individual differences of our human donors are minimized. The plot shows a clear separation in microbial composition between mice^ADHD^ and mice^control^ and clustering of the human donors (Additional file 1: figure S5). This clustering in the mice was also observed, though with less variation, after we removed the human samples and created a weighted average of the several collected samples (figure 1B). This means that averaging the samples of the different timepoints reduced the variation originating from (re-)colonization, although the same effect of groups was seen. Additionally, most (23 of the 31) of the defined genera were observed in both our human and animal samples.

*Cerebral blood flow*

Cerebral blood flow (CBF) was analyzed using FAIR-ASL. Three mice^control^ and two mice^ADHD^ were excluded from the analyses due to motion and/or echo planar imaging artifacts. Values from both hemispheres were averaged because no differences were detected in CBF between the right and left hemispheres of the corresponding ROI among the mouse groups. We detected no differences in CBF in the brain regions assessed between mice^control^ and mice^ADHD^.

*Total correlation analyses*

To compare functional connectivity (FC) patterns resulting from different microbial composition, rsfMRI data were statistically analyzed based on total and partial correlation in twelve ROIs: left and right dorsal hippocampus (DH), left and right ventral hippocampus (VH), left and right auditory cortex (AU), left and right motor cortex (M1), left and right somatosensory cortex (S1) and left and right visual cortex (V1). We found no microbiota-induced effects in the total correlations.

*Immunohistochemical stainings*

We visualized immature neurons with a polyclonal antibody against doublecortin (DCX) to investigate whether specific proteins connected to neurogenesis and synaptogenesis were altered in mice^ADHD^ as well. As a measure for neurogenesis, DCX-positive cells were counted in the subgranular zone of the hippocampus. We detected no differences in neurogenesis in the hippocampus. Postsynaptic density was stained with a polyclonal antibody against postsynaptic density protein 95 (PSD-95). Postsynaptic density was measured in the cortex and caudate putamen (Cpu). No statistically significant differences were detected in the cortex and Cpu between mice^control^ and mice^ADHD^.

**References**

1. Giles JM, Whitaker JW, Moy SS, Fletcher CA: **Effect of Environmental Enrichment on Aggression in BALB/cJ and BALB/cByJ Mice Monitored by Using an Automated System.** *Journal of the American Association for Laboratory Animal Science : JAALAS* 2018.

2. Pernold K, Iannello F, Low BE, Rigamonti M, Rosati G, Scavizzi F, Wang J, Raspa M, Wiles MV, Ulfhake B: **Towards large scale automated cage monitoring - Diurnal rhythm and impact of interventions on in-cage activity of C57BL/6J mice recorded 24/7 with a non-disrupting capacitive-based technique.** *PloS one* 2019, **14:**e0211063.

3. Etchebehere C, Tiedje J: **Presence of two different active nirS nitrite reductase genes in a denitrifying Thauera sp. from a high-nitrate-removal-rate reactor.** *Appl Environ Microbiol* 2005, **71:**5642-5645.

4. Lane DJ: **16S/23S rRNA sequencing. Nucleic acid techniques.** In *In Bacterial Systematics.* Edited by Stackebrandt E, and Goodfellow M. Chichester, UK: Wiley & Sons; 1991: 115-175

5. Daims H, Bruhl A, Amann R, Schleifer KH, Wagner M: **The domain-specific probe EUB338 is insufficient for the detection of all Bacteria: development and evaluation of a more comprehensive probe set.** *Syst Appl Microbiol* 1999, **22:**434-444.

6. van den Bogert B, de Vos WM, Zoetendal EG, Kleerebezem M: **Microarray analysis and barcoded pyrosequencing provide consistent microbial profiles depending on the source of human intestinal samples.** *Appl Environ Microbiol* 2011, **77:**2071-2080.

7. Ramiro-Garcia J, Hermes GDA, Giatsis C, Sipkema D, Zoetendal EG, Schaap PJ, Smidt H: **NG-Tax, a highly accurate and validated pipeline for analysis of 16S rRNA amplicons from complex biomes**  *F1000Research* 2016, **5:1791**

8. Morris EK, Caruso T, Buscot F, Fischer M, Hancock C, Maier TS, Meiners T, Muller C, Obermaier E, Prati D, et al: **Choosing and using diversity indices: insights for ecological applications from the German Biodiversity Exploratories.** *Ecology and evolution* 2014, **4:**3514-3524.

9. Wiesmann M, Zerbi V, Jansen D, Lutjohann D, Veltien A, Heerschap A, Kiliaan AJ: **Hypertension, cerebrovascular impairment, and cognitive decline in aged AbetaPP/PS1 mice.** *Theranostics* 2017, **7:**1277-1289.

10. Zerbi V, Wiesmann M, Emmerzaal TL, Jansen D, Van Beek M, Mutsaers MP, Beckmann CF, Heerschap A, Kiliaan AJ: **Resting-state functional connectivity changes in aging apoE4 and apoE-KO mice.** *J Neurosci* 2014, **34:**13963-13975.

11. Kim SG: **Quantification of relative cerebral blood flow change by flow-sensitive alternating inversion recovery (FAIR) technique: application to functional mapping.** *Magn Reson Med* 1995, **34:**293-301.

12. Kwong KK, Chesler DA, Weisskoff RM, Donahue KM, Davis TL, Ostergaard L, Campbell TA, Rosen BR: **MR perfusion studies with T1-weighted echo planar imaging.** *Magn Reson Med* 1995, **34:**878-887.

13. Paxinos G, Franklin KB: *The mouse brain in stereotaxic coordinates / George Paxinos, Keith Franklin.* London :: Academic; 2004.

14. Zerbi V, Jansen D, Wiesmann M, Fang X, Broersen LM, Veltien A, Heerschap A, Kiliaan AJ: **Multinutrient diets improve cerebral perfusion and neuroprotection in a murine model of Alzheimer's disease.** *Neurobiol Aging* 2014, **35:**600-613.

15. Harsan LA, Paul D, Schnell S, Kreher BW, Hennig J, Staiger JF, von Elverfeldt D: **In vivo diffusion tensor magnetic resonance imaging and fiber tracking of the mouse brain.** *NMR Biomed* 2010, **23:**884-896.

16. Zerbi V, Kleinnijenhuis M, Fang X, Jansen D, Veltien A, Van Asten J, Timmer N, Dederen PJ, Kiliaan AJ, Heerschap A: **Gray and white matter degeneration revealed by diffusion in an Alzheimer mouse model.** *Neurobiol Aging* 2013, **34:**1440-1450.

17. Tang Z-Z, Chen G, Alekseyenko AV: **PERMANOVA-S: association test for microbial community composition that accommodates confounders and multiple distances.** *Bioinformatics (Oxford, England)* 2016, **32:**2618-2625.
