# Supplementary material for: Gut microbiota from persons with attention-deficit/hyperactivity disorder affects the brain in mice
Source: Microbiome. 2020 Apr 1;8:44. doi: 10.1186/s40168-020-00816-x (PMC7114819; doi:10.1186/s40168-020-00816-x)
Supplement: Supplementary file 4 — Additional file 3: Title of data: Supplementary tables. Table S1. Imaging parameters for the anatomical references, rsfMRI, ASL and DTI. Table S2. Statistical outliers which were removed from the dataset per test. [file 40168_2020_816_MOESM3_ESM.docx]

**Table S1. Imaging parameters for the anatomical references, rsfMRI, ASL and DTI**

|  | Anatomical T2*weighted | rsfMRI | ASL | DTI |
| --- | --- | --- | --- | --- |
| Imaging method | GE | 4-shot Spin-echo EPI | FAIR-ASL | 6-shot spin-echo EPI |
| Echo time (ms) | 7.357 | 10 | 10.08 | 21 |
| Repetition time | 865.086 ms | 1.8 s | 12 s | 7.75 s |
| Image matrix | 512 × 512 | 96 × 96 | 128 × 128 | 128 × 128 |
| Field-of-view (mm) | 40 × 40 | 25 × 25 | 25 × 25 | 20 × 20 |
| Spatial resolution (μm/pixel) | 78 × 78 × 500 | 260 × 260 × 500 | 260 × 260 × 1000 | 156 × 156 × 500 |
| No. of slices bregma | 20 × 3 | 20 | 1 | 20 |
| Total acquisition time (min) | ∼8 | ∼11 | ∼13 | ∼35 |

**Table S2. Statistical outliers which were removed from the dataset per test**

| Test | Statistical outliers per experimental group | | Reason |
| --- | --- | --- | --- |
|  | Mice^ADHD^ | Mice^Control^ |  |
| Open Field Test (OFT) | 1 | 1 | Camera failure and jumping out of the arena |
| Novel Object Recognition (NOR)  (30 min interval) | 1 | 2 | Exploration bias (>75% of total exploration time exploring one object) |
| Novel Object Recognition (NOR)  (60 min interval) | 1 | 0 | Exploration bias (>75% of total exploration time exploring one object) |
| Diffusion tensor imaging (DTI) | 1 | 2 | Scans showed motion and/or echo planar imaging artifacts |
| Cerebral blood flow (CBF) | 2 | 3 | Scans showed motion and/or echo planar imaging artifacts |
| Resting-state fMRI (rsfMRI) | 4 | 5 | Scans showed motion and/or echo planar imaging artifacts |
